# Dissection of *in vivo* archaeal RNA polymerase assembly reveals a modular pathway and stoichiometric control

**DOI:** 10.1128/mbio.01440-26

**Published:** 2026-07-28

**Authors:** Jie Li, Wen Qi, Huaping Duan, Derong Ren, Jingfang Liu, Xiuzhu Dong

**Affiliations:** 1 State Key Laboratory of Microbial Diversity and Innovative Utilization, Institute of Microbiology, Chinese Academy of Sciences, Beijing, China; 2 University of Chinese Academy of Sciences 74519, Beijing, China; 3 Institutional Center for Shared Technologies and Facilities of Institute of Microbiology, Chinese Academy of Sciences, Beijing, China; Albert-Ludwigs-Universitat Freiburg, Freiburg, Germany

**Keywords:** RNA polymerase, archaea, protein assembly, transcription, stoichiometry, molecular chaperones

## Abstract

Archaeal transcription is driven by a multi-subunit RNA polymerase (RNAP) structurally and functionally homologous to eukaryotic RNAP II. However, its *in vivo* biogenesis remains uncharacterized. Here, we present the first comprehensive dissection of archaeal RNAP assembly using *Methanococcus maripaludis* as a genetically tractable model. Through an integrative strategy combining size exclusion chromatography, native purification, Western blotting, mass spectrum, and *in vitro* disassembly, we delineate a modular and hierarchical assembly pathway comprising five major subcomplexes. A stable Rpo3-11-2′2″ subcomplex was resolved by *in vitro* disassembly analysis, supporting a directional assembly route. Quantitative analyses revealed that RNAP subunits are maintained at balanced intracellular levels, and disruption of this stoichiometry, particularly overexpression of small subunits Rpo12 and Rpo11, disrupted RNAP assembly, reduced holoenzyme formation, and impaired growth. Rpo12 overexpression was associated with increased Rpo3/12 subcomplex, whereas Rpo11 overexpression caused accumulation of an Rpo3-11-10 intermediate; both coincided with reduced downstream assembly and holoenzyme formation, underscoring the importance of balanced subunit abundance for productive RNAP assembly. Moreover, mass spectrum identified chaperonin Hsp60 consistently co-eluting with RNAP subcomplexes, implying it as a candidate assembly cofactor. Together, these findings establish an *in vivo* framework for archaeal RNAP assembly, revealing a modular and hierarchical biogenesis pathway governed by stoichiometry-sensitive checkpoints. Given the shared subcomplex architecture and assembly logic, these insights not only fill a longstanding knowledge gap in archaeal RNAP assembly but also illuminate its evolutionary continuity with bacterial and eukaryotic systems and uncover regulatory vulnerabilities with potential applied relevance.

**IMPORTANCE:**

RNA polymerase is the core transcriptional machinery that underpins gene expression across domains of life, yet the *in vivo* assembly of the multi-subunit RNAP in archaea has remained poorly understood. This study provides the first *in vivo* dissection of archaeal RNA polymerase assembly, uncovering a modular and evolutionarily conserved pathway whose progression depends on balanced subunit stoichiometry. Imbalance of small subunits selectively disrupts early assembly steps, limits holoenzyme formation, and compromises cellular growth, establishing assembly fidelity as an intrinsic regulatory layer of archaeal transcription. By defining conserved assembly logic and stoichiometry-dependent checkpoints, this work fills a major gap in archaeal transcription biology, uncovers mechanistic parallels with bacterial and eukaryotic systems, and highlights RNA polymerase assembly as a previously unrecognized vulnerability in methanogenic archaea with potential relevance to methane mitigation.

## INTRODUCTION

As the central conduit of genetic information, gene expression orchestrates nearly all cellular processes, shaping physiology, adaption, and survival across all domains of life (1, 2). The first and most tightly regulated step in gene expression is transcription, in which RNA is synthesized from a DNA template by RNA polymerase (RNAP). Although single-subunit RNAPs exist in certain viruses (e.g., T7 phage) and organelles, most cellular RNAPs are hetero-multimeric complexes that share a conserved catalytic architecture (3, 4). Proper assembly of this multimeric complex is a prerequisite for accurate transcription, and defects in RNAP biogenesis can compromise gene expression and impair growth and cellular survival (3–6). Despite their essentiality to gene expression, the principles and pathways underlying RNAP biogenesis *in vivo* remain incompletely understood, especially in archaea.

Multi-subunit RNAPs are evolutionarily related, and this is reflected in their structure and subunit composition (3, 4). Bacterial RNAPs are comparatively simple, consisting of a five-subunit catalytic core (β″, β′, α′, α″, and ω) (7–9), while most eukaryotes possess three nuclear RNAPs (I, II, and III), each specialized for transcribing different gene classes, with RNAP I transcribing rRNA genes, RNAP II synthesizing mRNAs and noncoding RNAs, and RNAP III transcribing tRNAs and other small RNAs (4, 10). Archaea utilize a single RNAP that is structurally and functionally similar to eukaryotic RNAP II, consisting of 10–12 subunits and requiring basal transcription factors such as TATA-binding protein (TBP) and transcription factor B (TFB), orthologous to eukaryotic TBP and TFIIB (11–13). This shared architecture makes archaeal RNAPs as a powerful model system for understanding both ancestral and derived features of RNAP activity and its biogenesis. Classical studies in bacteria and subsequent work on eukaryotic RNA polymerase II established the view that multisubunit RNAP biogenesis proceeds through ordered, modular assembly intermediates. In bacteria, α-subunit dimerization nucleates an assembly platform that subsequently recruits β and β′ to build the catalytic core, whereas studies of Pol II suggested a related assembly logic for the universally conserved subunits within a more elaborate eukaryotic framework (5, 6, 8). However, whether and how this conserved assembly principle operates in archaeal RNAP *in vivo* has remained poorly defined.

Archaea occupy a pivotal phylogenetic position bridging bacteria and eukaryotes (14, 15). They thrive in diverse environments, from extreme environments to soils, oceans, freshwater, and host-associated microbiomes (16–19), underscoring their ecological importance and robust roles in driving global biogeochemical cycles. Phylogenomic advances, particularly the discovery of the Asgard superphylum (20, 21), have further revealed that eukaryotes likely emerged from within the archaea domain with Asgard lineages representing the closest known archaeal relatives of eukaryotes. Mirroring this evolutionary position, archaeal genomes combine bacterial-like features such as compact genomes, polycistronic operons, and short intergenic regions, with eukaryotic-like machineries for replication, transcription, and translation (22). This evolutionary mosaic renders archaea uniquely suited to dissect the molecular principles of RNAP biogenesis and transcriptional regulation and to illuminate the evolutionary transition from prokaryotic simplicity to eukaryotic complexity.

Structural studies have established the architecture of archaeal RNAP, centered on two large subunits Rpo1 and Rpo2 (often split into 1′/1″ and 2′/2″ in many species), which harbor evolutionarily conserved double-psi β-barrel (DPBB) motifs (3, 13, 23, 24). Additional subunits are organized into conserved accessory modules that stabilize the core complex and support transcriptional function. These include the Rpo3-11-10-12 module (orthologous to eukaryotic Rpb3, Rpb11, Rpb10, and Rpb12), Rpo6 and Rpo5 (homologs of eukaryotic Rpb6 and Rpb5), and the stalk subcomplex Rpo4 and Rpo7 (analogous to Rpb4/7) (Table S1). Although structural and biochemical studies have provided detailed insights into the architecture and catalytic activity of archaeal RNAP, the mechanisms by which these subunits are assembled *in vivo* into a functional enzyme remain poorly defined.

To date, most knowledge about archaeal RNAP assembly has been derived from *in vitro* reconstitution experiments, particularly using recombinant subunits from *Methanocaldococcus jannaschii*. These studies suggest a stepwise assembly process initiated by formation of the Rpo3-11-10-12 scaffold, which facilitates the recruitment and stabilization of the catalytic subunits (Rpo 1′/1″, 2′/2″) and accessory modules (25, 26). For example, the Rpo3-11-10-12 subcomplex was shown to enhance solubility of catalytic subunits *in vitro*, enabling minimal RNAP (Rpo3/11/10/12-1′1″2′2″) activity (26). However, these observations lack validation under physiological conditions, and the regulatory mechanisms that coordinate RNAP biogenesis in living archaeal cells remain unknown. Our recent work uncovered a transcriptional control mechanism that links RNAP subunit expression to assembly control. In the methanogenic archaeon *Methanococcus maripaludis*, the small subunit Rpo12 is precisely regulated by an operon-internal transcription termination site (ioTTS). Genetic disruption of this ioTTS leads to Rpo12 overexpression and a concomitant reduction in the assembled RNAP complex and intermediates (27). These findings suggest that imbalanced expression of the small subunit can perturb RNAP assembly. Yet, the mechanism underlying this defect, and how it reflects the overall assembly pathway, remains to be elucidated.

Here, we present the first *in vivo* dissection of the archaeal RNAP assembly process, using *M. maripaludis* as a genetically tractable model archaeon that supports robust CRISPR-based manipulation and synthetic biology applications (28–32). Through a combination of size exclusion chromatography (SEC), native purification, subunit-specific Western blotting, and *in vitro* disassembly assays, we delineate a modular and hierarchical assembly cascade comprising five major subcomplexes. Absolute quantification of RNAP subunits revealed tightly regulated intracellular stoichiometry, and perturbations to this balance, specifically via overexpression of Rpo12 or Rpo11, disrupted discrete assembly steps, leading to impaired RNAP formation and reduced cell growth. Mass spectrometry further identified chaperon Hsp60 as a potential assembly cofactor. Together, these findings establish a mechanistic framework for archaeal RNAP biogenesis, highlight its evolutionary continuity with bacterial and eukaryotic systems, and suggest potential vulnerabilities in methanogenic archaea relevant to methane mitigation strategies.

## RESULTS

### Identification of *in vivo* archaeal RNAP assembly subcomplexes via size exclusion chromatography and Western blotting

**Fig 1:**
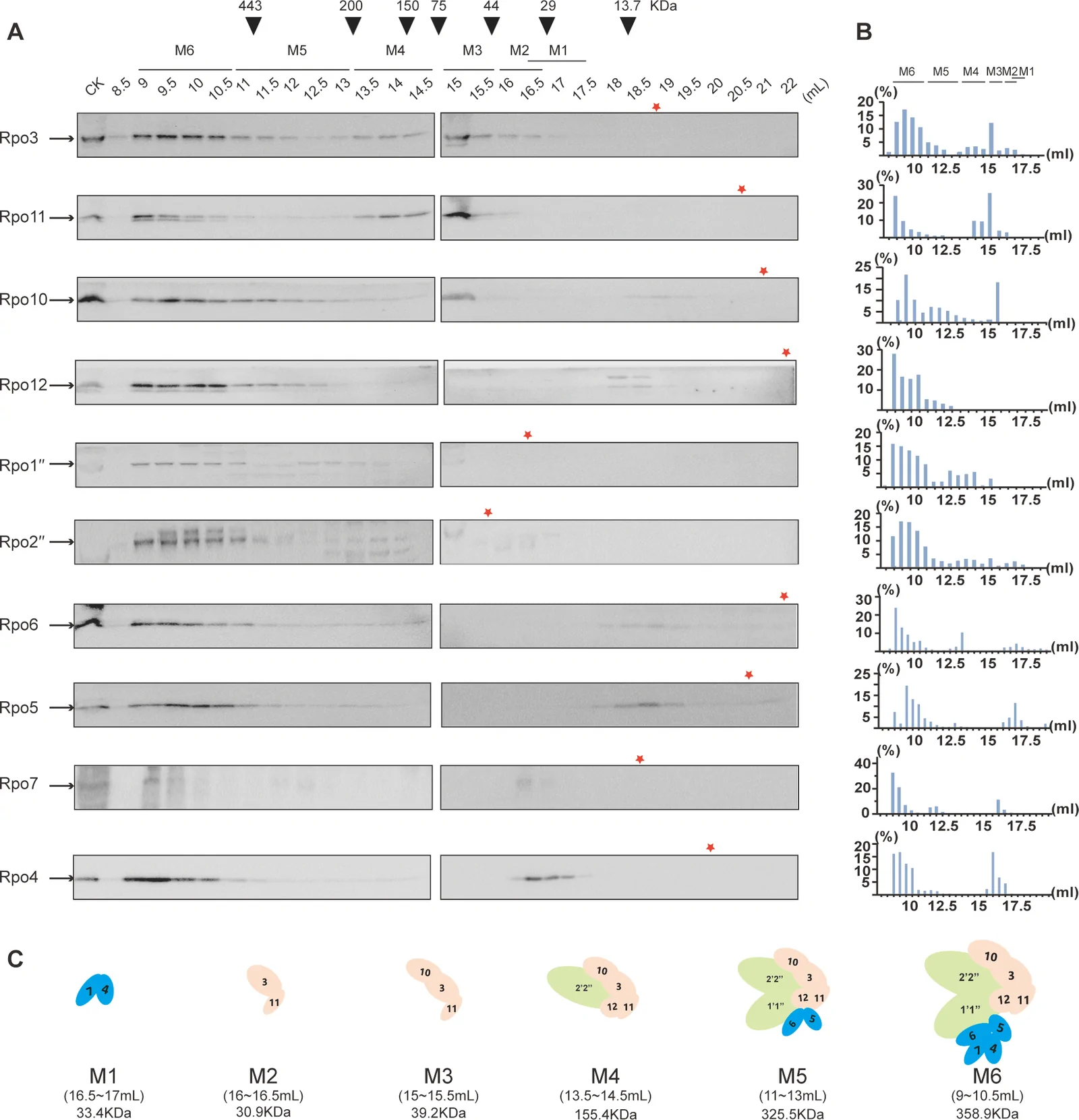
Identification of the *in vivo* RNAP assembly intermediates in *M. maripaludis* by size-exclusion chromatography (SEC) and subunit-specific Western blotting. (**A, B**) Western blotting was performed on SEC fractions (from 8.5 to 22 mL) using antibodies against 10 individual RNAP subunits. Cell-free extract of *M. maripaludis* was fractionated by SEC. Representative immunoblots and corresponding quantitative bar charts show the distribution of RNAP subunits across fractions. RNAP subcomplexes and fully assembled complex (M1–M6) are indicated above the gel by reference to the SEC profiling combined with Western blotting. The fractions where the molecular markers eluted are indicated by inverted triangles at the top. CK indicates the control lane to determine the target position by loading with the cell-free extract. Letters flanking arrows indicate the specific subunit targeted by each antibody against the subunit. Red stars indicate the theoretical elution volume (mL) (Table S2) of individual RNAP subunits at free form, calculated based on the SEC calibration curve (Fig. S1). (**C**) Schematic diagram of defined RNAP assembly subcomplexes (M1–M6) based on SEC elution profiles combined with Western blotting detection. M1 to M5 represent successive intermediates in the RNAP assembly cascade; M6 corresponds to the fully assembled RNAP holoenzyme.

To investigate the *in vivo* assembly pathway of archaeal RNAP, we employed *M. maripaludis*, a fast-growing model archaeon with a robust genetic system and well-developed genetic toolkits, including CRISPR-Cas-based genome editing and synthetic biology elements (28–31). Cell-free extracts from logarithmic phase cultures were fractioned by size exclusion chromatography (SEC) on a Superdex 200 column into 48 fractions (0.5 mL each). A calibration curve was generated using seven marker proteins of known molecular weights (Fig. S1), enabling estimation of the apparent molecular size of proteins in each fraction (annotated at the top of Fig. 1). To monitor the distribution of RNAP subunits across the SEC fractions, 10 subunits (Rpo3, 11, 10, 12, 1″, 2″, 5, 6, 7, and 4) were heterogeneously expressed and purified in *E. coli* and used to generate polyclonal antisera for Western blotting. Subunit-specific immunoblotting of SEC fractions ranging from 8.5 to 22 mL revealed distinct distribution profiles, indicative of both free subunits and assembled RNAP (sub)complexes. Most subunits did not accumulate at their theoretical monomeric positions (predicted from the calibration curve), indicating that majority exist in assembled or partially assembled forms (Fig. 1A and Table S2). Only weak signals corresponding to free subunits (<5% of total abundance) were observed at ≥18.5 mL (Fig. 1B), particularly for small subunits Rpo3, Rpo10, and Rpo5. Analysis of SEC and Western blotting profiles revealed five major RNAP subcomplexes, designated M1 to M5, eluting in a stepwise manner from higher to lower elution volumes. M1 (16.5–17 mL) contained Rpo7 and Rpo4, and M2 (16–16.5 mL) included Rpo3 and Rpo11, consistent with their theoretical sizes (Fig. 1A and Table S3). Then, bigger subcomplex M3 (15–15.5 mL) could be composed by Rpo3, 11, and 10, with possible inclusion of Rpo12. Furthermore, bigger subcomplex M4 (13.5–14.5 mL) contained Rpo3, 11, 10, and 2′2″ subunits (Fig. 1 and Table S3). The larger subcomplex M5 (11–13 mL) was composed by Rpo3, 11, 10, 12, 2′2″, 1′1″, 5, and 6 subunits. Finally, the fully assembled RNAP holoenzyme (M6) was eluted at 9–10.5 mL, where all 12 subunits, including the Rpo4/7 heterodimer, co-eluted as a single complex (Fig. 1 and Table S3).

Western blotting band intensities were further quantified across SEC fractions to estimate the relative abundance within the defined (sub)complexes. Quantification determined that although discrete assembly intermediates (M1–M5) were detectable, they represented minor species compared to the assembled complex (M6), which accounted for ~50%–70% of total RNAP in logarithmic-phase extracts (Fig. 1B). For example, the abundances of Rpo3 and 11 subunits present in these (sub)complexes, M1 (16.5–17.5 mL), M2 (16–16.5 mL), M3 (15–15.5 mL), M4 (13.5–14.5 mL), M5 (11–13 mL), and M6 (9–10.5 mL), were 2.83%, 5.06%, 14.26%, 9.33%, 13.82%, and 55%, respectively (Fig. 1B and Table S4). Rpo10 exhibited in a distribution pattern similar to Rpo3, whereas Rpo2″ was mainly detected in M4–M6 (sub)complexes at 8.41%, 18.97%, and 59.70%, respectively (Fig. 1B and Table S4). Likewise, similar as Rpo1″, Rpo6 and Rpo5 were mainly present in M4–M6 (Fig. 1B). These findings suggest that archaeal RNAP assembles through an efficient and modular pathway, in which intermediates show limited steady-state accumulation relative to holoenzyme.

Together, these results define a modular *in vivo* assembly process consisting of five major intermediates, which could be M1 (Rpo4/7 stalk heterodimer), M2 (Rpo3/11), M3 (Rpo3/11/10, possibly including Rpo12), M4 (Rpo3/11/2′2″, possibly including Rpo12), M5 (possibly Rpo3/11/10/12/2′2″/1′1″/6/5), and M6 (the fully assembled RNAP complex). The low steady-state abundance of intermediates relative to M6 indicates that RNAP assembles through an efficient pathway, with transient intermediates that do not accumulate to high levels *in vivo*.

### Purification and compositional validation of RNAP subcomplexes from an Rpo11-tagged strain

**Fig 2:**
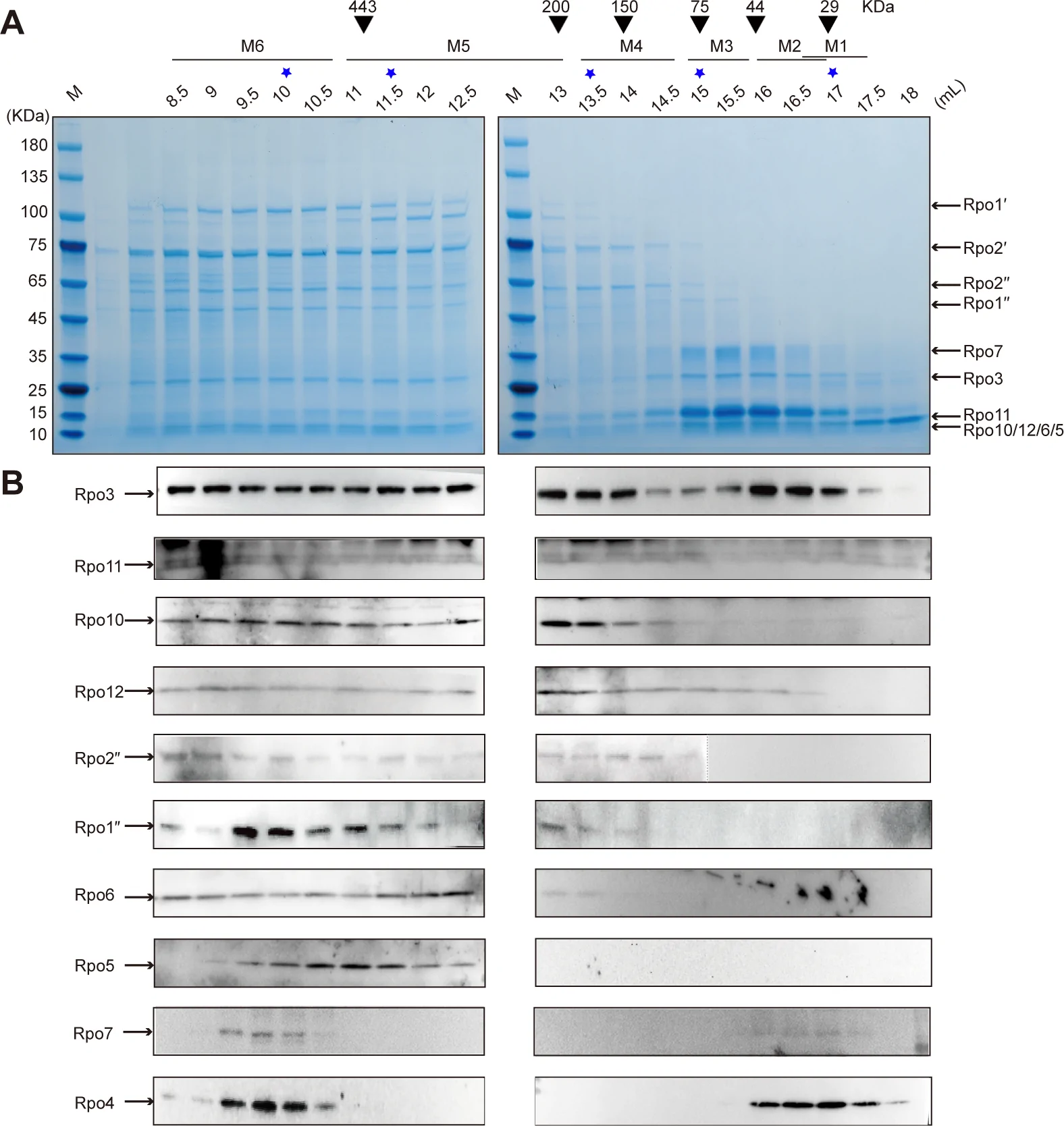
SDS-PAGE, Western blotting, and mass spectrometry analyses reveal the subunit composition of *M. maripaludis* RNAP assembly intermediates. (**A**) SDS-PAGE analysis of partially purified RNAP complexes from indicated SEC fractions (from 8.5 to 17.5 mL). The partially purified RNAP complexes were fractioned by SEC. RNAP subcomplexes and fully assembled complex (M1–M6) are indicated above the gel by reference to the SEC profiling combined with SDS-PAGE. The fractions where the molecular markers eluted are indicated by inverted triangles at the top. The RNAP subunits, determined by the molecular weights by reference to the protein markers, are indicated by arrows. The RNAP subcomplexes labeled above the Asterisks indicate the fractions subjected to LC–MS/MS analysis. (**B**) Western blotting of SEC fractions using subunit-specific antibodies to compare the distribution and relative abundance of RNAP subunits across the SEC fractions of partially purified RNAP (sub)complexes. M1 to M6 denote sequential assembly intermediates, with M6 representing the fully assembled holoenzyme.

Next, to validate the subcomplexes identified from cell-free extract (CFE) analysis, we further purified the RNAP and its assembly intermediates from a *M. maripaludis* strain expressing a chromosomally His10×-tagged Rpo11 (see Materials and Methods), a small and initially assembled subunit enabling efficient capture of RNAP (sub)complexes. Partial purification under non-stringent Ni-NTA affinity chromatography conditions enriched both the fully assembled RNAP and partially assembled subcomplexes. The eluates were further fractionated by SEC on a Superdex 200 column analyzed in parallel by SDS-PAGE and subunit-specific Western blotting (Fig. 2A and B). Based on these results (Fig. 2A and B), it was demonstrated that in the fractions of 16.5 to 17.5 mL, Rpo4/7 heterodimeric subcomplex (M1) was detected, while fractions of 16 to 16.5 mL contained Rpo3/11/12 (M2) subcomplex, possibly including Rpo10, consistent with the CFE result (Fig. 1). In the fractions of 15.5–15 mL (M3) and 14.5–13.5 mL (M4), it could be detected that Rpo2″ and Rpo2′ were successively recruited to the Rpo3/11/10/12 scaffold. Based on comparative abundances, Rpo2″ appeared to be recruited prior to Rpo2′.

Rpo1′1″ was detected beginning at 13.5 mL, following incorporation of Rpo2′2″ (Fig. 2A). The small subunits Rpo6 and Rpo5 appeared in fractions 11–12.5 mL (M5) (Fig. 2B), consistent with their incorporation during a similar late stage of RNAP assembly. Structural modeling suggests that Rpo6 and Rpo5 could bind directly to Rpo1′ (Fig. S2); however, the present data do not distinguish whether these subunits are recruited independently or as a pre-assembled subcomplex. Finally, after the recruitment of Rpo1′1″-6-5, the Rpo4/7 heterodimer could be largely detected beginning at 10.5 mL (Fig. 2B), coinciding with the appearance of the most complete assembly states. Correspondingly, the fully assembled RNAP holoenzyme (M6) with a major peak at 9.5–10 mL could be detected.

Therefore, these results reinforce the subcomplex identities observed in the CFE analysis, while providing additional resolution on subunit recruitment order. Notably, they support the possible co-occurrence of the M3 (Rpo3/11/10/12) and M4 subcomplexes (Rpo3/11/10/12/2′2″) in partially overlapping fractions (14.5–15 mL). In later fractions, the concomitant appearance of Rpo1′1″ with Rpo5/6 suggests that these subunits are recruited during a similar late assembly window, although the present data do not distinguish whether they are incorporated independently or as a pre-assembled module.

### Mass spectrum validation of RNAP subcomplex composition and cofactors

To independently validate the composition of RNAP subcomplexes (M1 to M6), proteins in selected SEC fractions were analyzed by LC-MS/MS. In fractions corresponding to M1/M2 fraction (16.5 mL), Rpo3/11 were reproducibly detected (Fig. S3 and Data sets S1 through S4). In the M3 fraction (15 mL), both Rpo3/11 and Rpo2″2′ were detected, consistent with the presence of Rpo2″2′ in this fraction detected in the SDS-PAGE analysis (Fig. 2A), and confirming the recruitment of catalytic subunits Rpo2″2′ to the Rpo3/11 platform. In the M4 fraction (13.5 mL), the Rpo3/11/10/12 and the catalytic subunits of Rpo2″2′ and Rpo1″1′ were detected, indicating further progression along the assembly pathway. Furthermore, all these subunits could be detected in the M5 fraction (11.5 mL) by MS. In the M6 fraction (10 mL), the broadest complement of RNAP subunits, including Rpo3/11/10/12, catalytic subunits Rpo2″2′1″1′, and small subunits Rpo6, was identified by MS. Therefore, the MS assay further verified the subunit compositions of each identified RNAP subcomplexes determined above.

Then, we also analyzed the possible archaeal RNAP assembly cofactors determined by the MS assay. In addition to RNAP subunits, MS consistently identified the archaeal group II chaperonin GroEL (Thermosome, HSP60 family) across the fractions of M3 to M6 (Data sets S1 through S4), suggesting its potential involvement in subsequent RNAP assembly following the Rpo3/11 assembly, possibly by assisting in RNAP subunit folding or complex stabilization. This finding suggests that this chaperonin is likely involved in archaeal RNAP assembly process.

### Disassembly of the purified RNAP complex reveals the minimal stable RNAP subcomplex

**Fig 3:**
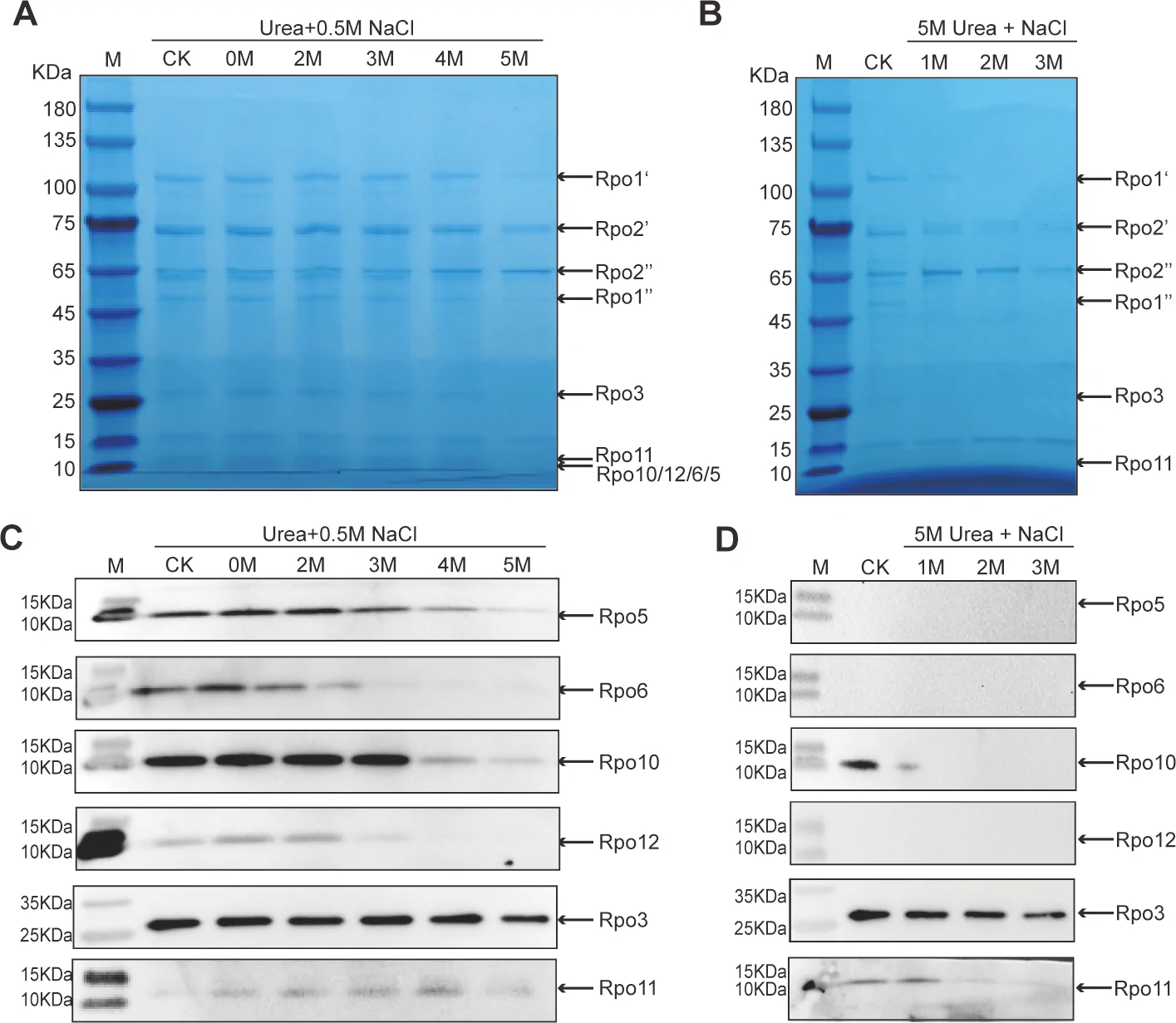
*In vitro* disassembly of *M. maripaludis* RNA polymerase (RNAP) subunits under urea and high-salt conditions. (**A and B**) SDS-PAGE analysis of RNAP subunits released upon treatment with increasing concentrations of urea. The concentrations of urea and NaCl used in each assay are indicated at the top of the gel. In panel A, samples were treated with increasing urea concentrations in the presence of 0.5 M NaCl. In panel B, samples were treated with 5 M urea and the indicated NaCl concentrations. M, the protein makers with indicated molecular weights. CK, the control reaction without urea treatment. (**C and D**) Western blotting detected RNAP subunit dissociation under combined urea and high-salt conditions. The concentrations of urea and NaCl used in each assay are indicated above the blots. RNAP subunits detected by subunit-specific antibodies are indicated by arrows.

To further probe the stability and modular organization of RNAP subcomplexes, we subjected the purified RNAP holoenzyme to *in vitro* disassembly assays under denaturing conditions. Highly purified RNAP was obtained from the Rpo11-His10 × tagged *M. maripaludis* strain using a strict purification procedure (Fig. S4). *In vitro* disassembly was initially induced under mild denaturing conditions by increasing concentrations of urea (0 to 5 M) in a buffer containing 0.5 M NaCl. As shown in Fig. 3A and B, the RNAP small subunits except Rpo3/11 were gradually dissociated from the catalytic subunits Rpo1′1″2′2″ as the urea concentration increased, and the catalytic subunits Rpo1′1″ also began to release under 5 M urea condition. Subsequently, to further destabilize the complex, we treated the purified RNAP complex under more stringent denaturing conditions by adding 1 M, 2 M, and 3 M NaCl to a buffer containing 5 M urea. As shown in Fig. 3C and D, under these harsher conditions, the catalytic subunits Rpo1′1″ were further dissociated, while Rpo2′2″ remained tightly associated with Rpo3 and Rpo11.

Notably, even under the most stringent condition (5 M urea + 3 M NaCl), Rpo2′2″ did not dissociate from Rpo3/11 (Fig. 3C and D), suggesting that the Rpo3/11–Rpo2′2″ complex likely constitutes the minimal stable subassembly of archaeal RNAP. These findings support a model in which Rpo3/11 not only serves as an initial assembly platform but also could be the minimal unit capable of directly recruiting and stabilizing the catalytic core subunits Rpo2′ and 2″.

### Quantitative analysis reveals stoichiometric balance among RNAP subunits in *M. maripaludis*

Our previous study demonstrated that the expression of *rop12* is regulated by an internal transcription termination site (ioTTS) and that disruption of this ioTTS element leads to Rpo12 overexpression and a concomitant reduction in both fully assembled RNAP and its assembly intermediates (27). These findings suggested a possible stoichiometric control mechanism underlying RNAP biogenesis. To investigate whether Rpo12 acts as a limiting factor for assembly under physiological conditions, we performed quantitative Western blotting to estimate the cellular abundance of the RNAP subunits in *M. maripaludis*.

**Fig 4:**
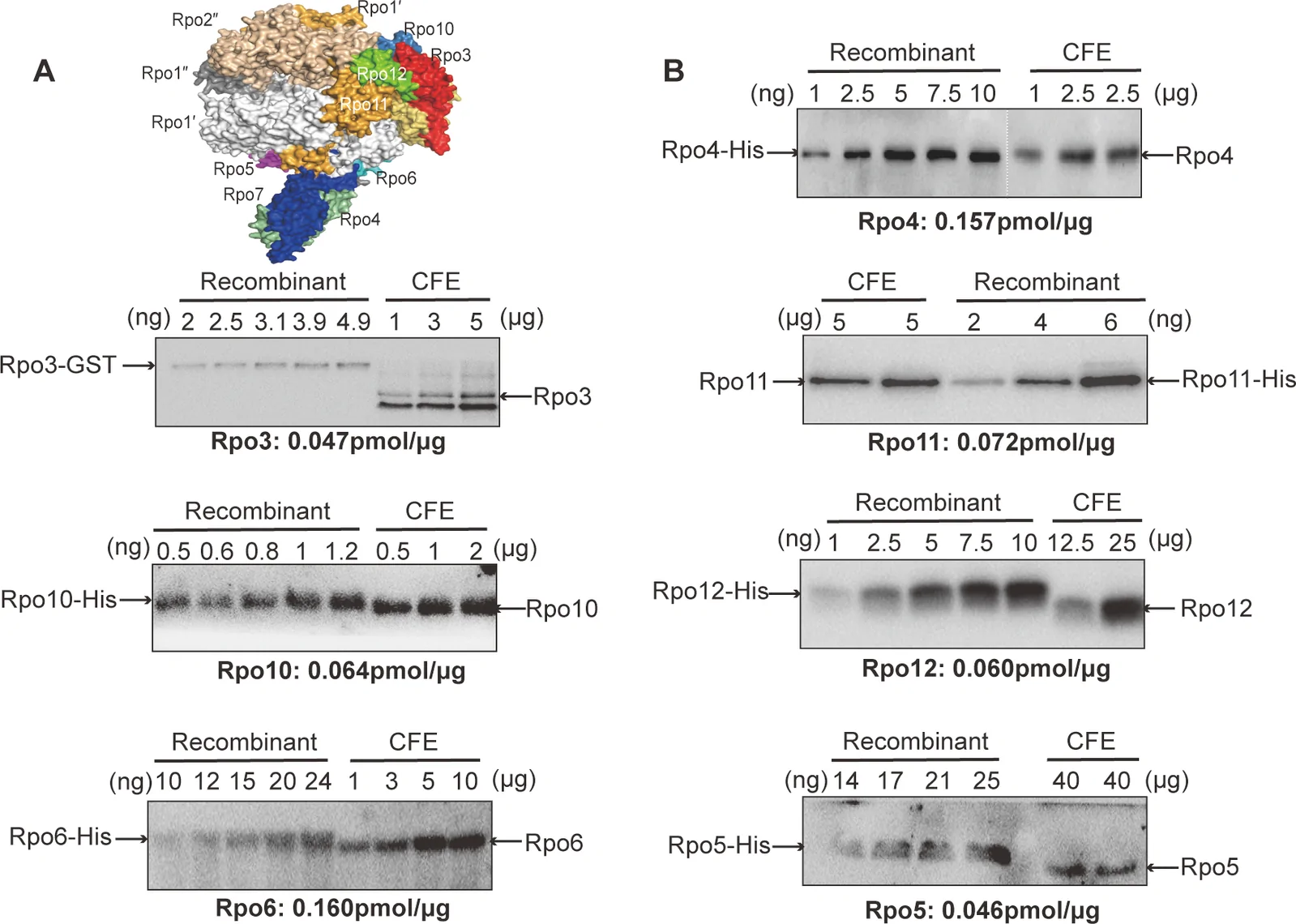
Quantitative analysis of RNAP subunit abundances in *M. maripaludis*. (**A**) Structure of the fully assembled RNAP holoenzyme predicted by AlphaFold. The RNAP subunits are indicated by different colors. (**B**) Quantitative Western blotting analysis of RNAP subunit abundances in the wild-type strain. Cell-free extracts (CFE) were probed with subunit-specific antibodies, and band intensities were compared to standard curves generated using purified recombinant RNAP subunits (left lanes; see also Fig. S4). The determined abundance of each RNAP subunit in CFE is indicated below each corresponding gel panel.

The RNAP subunits (Rpo3, 11, 10, 12, 5, 6, 4, and 7) were recombinantly expressed in *E. coli*, purified, and used to establish standard curves by serial dilutions for quantitative Western blotting. Whole cell-free extracts from logarithmic-phase *M. maripaludis* cultures were then probed with the subunit-specific antibodies to determine the cellular abundance of each tested RNAP subunit. Based on this quantifying method, the cellular abundances of Rpo3, 11, 10, and 12 were determined at comparable levels, ranging from 0.047 to 0.072 pmol/µg total proteins (Fig. 4 and Fig. S5). Rpo6 and Rpo7 were moderately more abundant (0.16 and 0.157 pmol/µg, respectively), while Rpo5 was present at a similar level (0.046 pmol/µg) (Fig. 4 and Fig. S5).

These data demonstrate that the tested RNAP subunits are maintained within a narrow and balanced concentration range (~0.05–0.15 pmol/µg) and that Rpo12 is not uniquely underrepresented and is unlikely to be a limiting factor for RNAP assembly under physiological conditions. Instead, the previously observed RNAP assembly defects upon Rpo12 overexpression likely reflect perturbations in subunit stoichiometry rather than natural insufficiency.

### Overexpression of several small RNAP subunits impairs the growth of *M. maripaludis*

Next, to assess whether imbalanced expression of other RNAP subunits affects cellular physiology, we systematically overexpressed selected RNAP subunits in *M. maripaludis* and investigated their impact on growth. The coding sequences, encoding Rpo1′1″, 2′2″, 11, 10, 6, 5, and 4/7, were individually cloned into the multicopy expression plasmid pMEV4 under a constitutive promoter, resulting in simultaneous expression from both plasmid and native genomic loci, as previously constructed for Rpo12 (27).

Growth phenotypes of the resulting overexpression strains were evaluated at both 37°C (optimal temperature) and 22°C (suboptimal temperature). At both temperatures, strains overexpressing Rpo11, Rpo5, or Rpo4/7 exhibited significantly reduced growth rates, like the phenotype previously observed upon Rpo12 overexpression (Fig. S6 and Fig. 7A). In contrast, the overexpression of the catalytic subunit Rpo2′2″ or small subunits Rpo10 and Rpo6 resulted in only minor growth defects. The overexpression of the catalytic subunit Rpo1′1″ had a mild impact on growth at 37°C and a slight effect at 22°C (Fig. S6 and Fig. 7A and B). Given that Rpo5 and Rpo4/7 are likely recruited during later stages of RNAP complex formation, the observed growth reduction upon their overexpression may not reflect the effects on RNAP assembly, which instead could be stem from other possible effects on RNAP activity alteration. In contrast, the growth defects associated with Rpo12 and Rpo11 overexpression attracted our attention. Both subunits are involved in the early stages of RNAP biogenesis and are determined to constitute part of the initial assembly scaffold, likely the Rpo3/11/10/12 subcomplex. Their overexpression may have a correlation with the compromising of RNAP assembly interference by disrupting subunit stoichiometry. These findings promoted us to further investigate the possible mechanistic linkage between the RNAP assembly and subunit stoichiometry.

### Overexpression of Rpo12 and Rpo11 perturbs RNAP assembly via different ways

To determine whether the growth defects caused by Rpo12 and Rpo11 overexpression correlate with disruptions of RNAP assembly, we compared the RNAP assembly profiles in the corresponding overexpression strains to those of the wild-type (WT) strain. SEC coupled with subunit-specific Western blotting was employed to resolve and quantify RNAP (sub)complexes in the overexpression and WT strains, and all samples were analyzed in parallel under identical chromatographic and detection conditions, allowing for direct comparison of each subunit’s abundance across SEC fractions.

**Fig 5:**
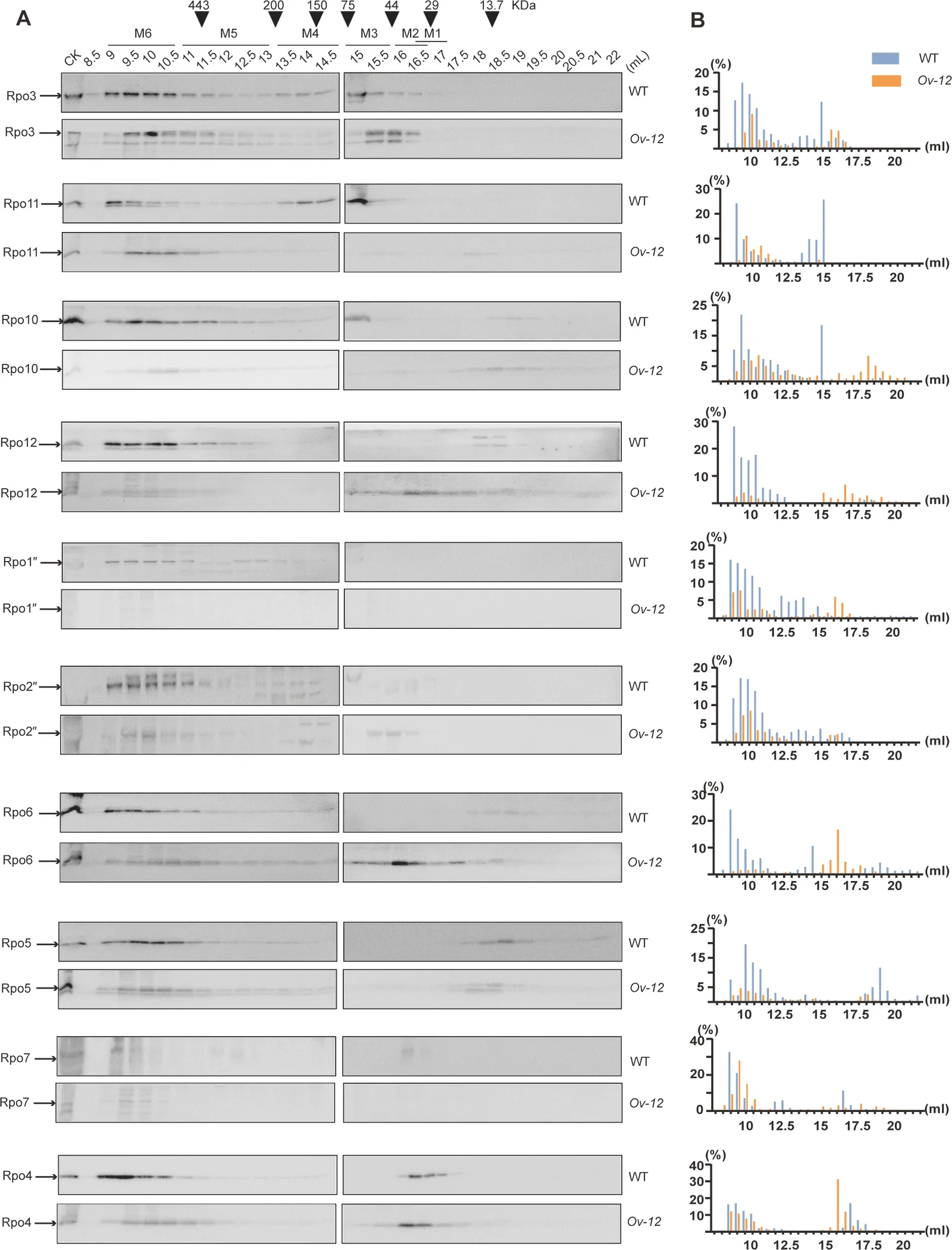
Overexpression of Rpo12 disrupts RNAP assembly in *M. maripaludis*. (**A**) Western blotting analysis of the SEC fractions from wild-type and Rpo12 overexpressing strains using subunit-specific antibodies. The SEC fractions, RNAP (sub)complexes (M1 to M6), and the SEC markers are all indicated similarly as in Fig. 1. WT and *Ov-12* indicate the wild-type and the Rpo12 overexpression strains. The WT SEC–Western blotting profiles shown in this figure are reproduced from the data set presented in Fig. 1 and are included here solely to facilitate direct comparison with the corresponding overexpression strain. (**B**) Bar chart showing the relative abundance ratios of RNAP subunits in each SEC fractions of the Rpo12-overexpressing strain (*Ov-12*) compared to the wild-type (WT) control, based on signal intensity from Western blotting quantification.

In the Rpo12 overexpression strain, we observed a global reduction in the abundance of all major RNAP assembly intermediates, including M3 (Rpo3/11/10, possibly including Rpo12), M4 (Rpo3/11/10/12–Rpo2′2″), M5 (Rpo3/11/10/12–Rpo2′2″–Rpo1′1″–6/5), and the fully assembled holoenzyme (M6: Rpo3/11/10/12–Rpo2′2″–Rpo1′1″–6/5/4/7) (Fig. 5). Strikingly, an Rpo3/12-containing subcomplex became prominent in fractions 15.5–16.5 mL, coinciding with a sharp reduction in the abundance of the native M2 (Rpo3/11) subcomplex. These changes suggest that excess Rpo12 alters the distribution of early Rpo3-containing assembly states and may interfere with downstream assembly progression, leading to reduced abundance of subsequent assembly intermediates and holoenzyme. Consistently, increased signals for Rpo12, Rpo10, and Rpo6 were detected in the later SEC elution fractions (≥18 mL) in the Rpo12 overexpression strain, suggesting that these subunits remained unassembled or dissociated from the RNAP complex.

**Fig 6:**
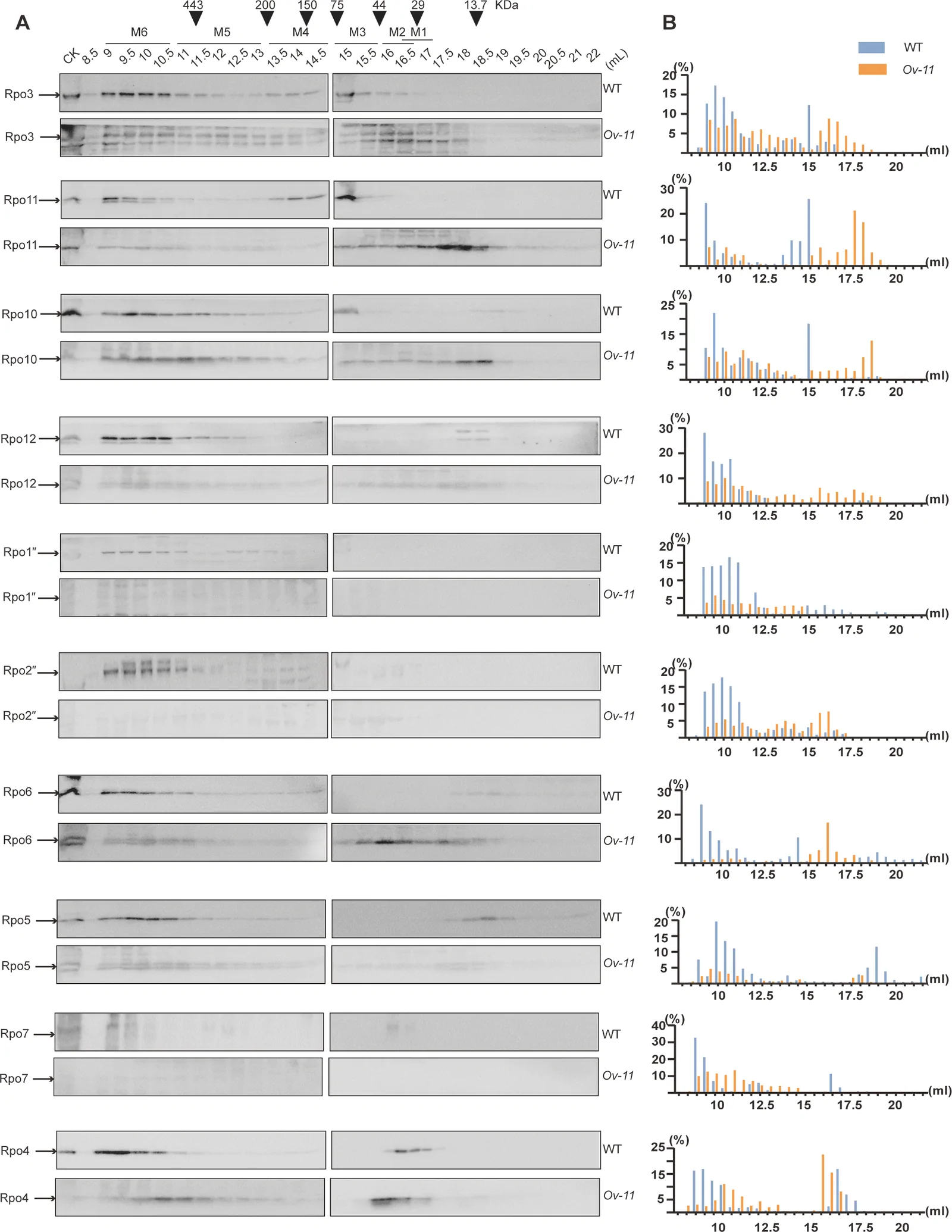
Overexpression of Rpo11 alters the distribution of RNAP assembly intermediates in *M. maripaludis*. (**A**) Western blotting analysis of SEC fractions from wild-type (WT) and Rpo11-overexpressing (*Ov-11*) strains using subunit-specific antibodies. The SEC fractions, RNAP (sub)complexes (M1 to M6), the SEC markers are all indicated similarly as in Fig. 1. WT and *Ov-11* indicate the wild-type and the Rpo11 overexpression strains. The WT SEC–Western blotting profiles shown in this figure are reproduced from the dataset presented in Fig. 1 and are included here solely to facilitate direct comparison with the corresponding overexpression strain. (**B**) Bar chart showing the relative abundance ratios of RNAP subunits in each SEC fractions of the Rpo11-overexpressing strain (*Ov-11*) compared to the wild-type (WT) control, based on signal intensity from Western blotting quantification.

Similarly, the overexpression of Rpo11 also caused a substantial reduction in the abundance of downstream intermediates (M4–M6) (Fig. 6). However, a key difference was observed: Rpo11 overexpression led to marked accumulation of the M3 subcomplex (Rpo3/11/10) across fractions 15–18 mL (Fig. 6). This accumulation suggests that excess Rpo11 may favor formation or stabilization of this early assembly intermediate. Nevertheless, the accumulation of M3 subcomplex (Rpo3/11/10) was accompanied by inversely reduced abundance of subsequent assembly intermediates and the fully assembled RNAP complex, suggesting that increased M3 abundance did not translate into enhanced downstream assembly. Thus, excess Rpo11 may perturb normal assembly-state distribution and create a bottleneck effect at or near the M3 stage, thus depleting productive progression toward holoenzyme formation.

Together, these results demonstrate that the overexpression of either Rpo12 or Rpo11 had caused the prevalent reduction of the RNAP assembly intermediates and the final assembled RNAP complex, albeit through distinct molecular modes, i.e., Rpo12 overexpression caused accumulation of Rpo3/12 subcomplex and depletion of the native Rpo3/11 intermediate, whereas Rpo11 overexpression led to accumulation of a potentially functional-aberrant Rpo3/11/10 intermediate. These perturbations in RNAP assembly likely underlie the growth reduction phenotypes observed in both strains.

### Overexpression of Rpo12 and Rpo11 broadly reduced the cellular abundance of RNAP subunits

**Fig 7:**
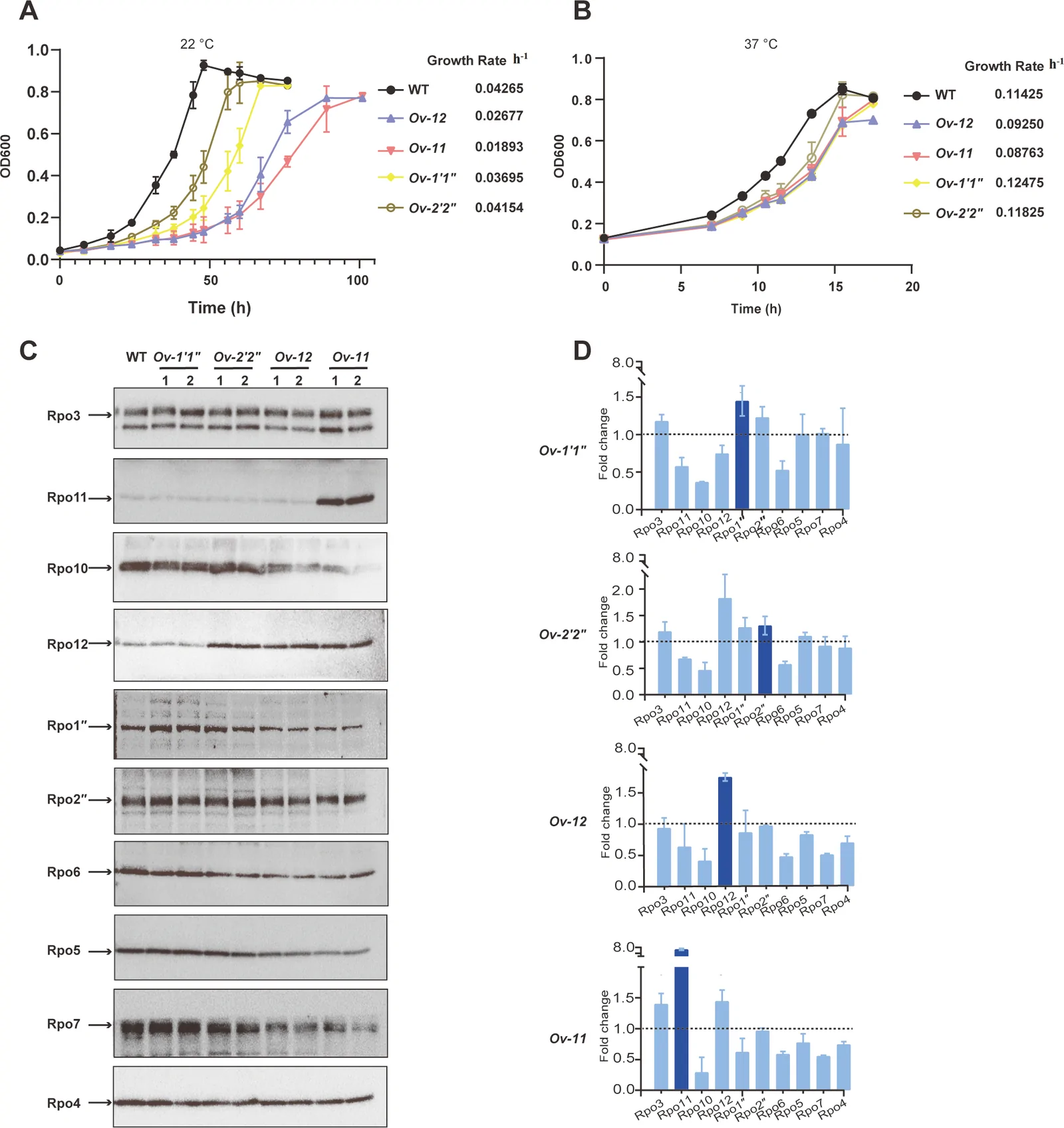
Impact of RNAP subunit overexpression on cell growth and RNAP subunit expression levels in *M. maripaludis*. (**A and B**) Growth curves of overexpression strains cultured at 22°C (**A**) and 37°C (**B**), compared to the wild-type (WT) strain. Growth curves represent mean ± SD from three independent biological replicates. (**C**) Western blotting analysis of RNAP subunit expression in each overexpression strain compared with the wild-type (WT) strain. Subunits overexpressed in each strain are indicated at the top of the panel; RNAP subunits detected by corresponding antibodies are indicated by arrows on the left. (**D**) Bar chart showing the relative abundance of each RNAP subunit in overexpression strains compared to the wild-type control, based on Western blotting signal intensity. *Ov-12*, *Ov-11*, *Ov-1′1″*, and *2′2″* indicate the *Rpo12*, *Rpo11*, *Rpo1′1″*, and *Rpo2′2″* subunit overexpression strains, respectively. Bars represent mean ± SD from at least three biological replicates.

To assess whether the overexpression of Rpo12 and Rpo11 affects the cellular abundance of RNAP components, we compared the total protein levels of RNAP subunits in whole-cell extracts of the respective overexpression strains with those of the wild-type (WT) strain. The Western blotting results revealed that, Rpo3, Rpo1″, and Rpo2″, which exhibited a modest reduction of ~20%, while most small RNAP subunits, including Rpo11, Rpo10, Rpo6, Rpo5, Rpo7, exhibited more pronounced decreases in protein abundance, ranging from ~30% to 70% (Fig. 7C and D). These data indicated that the overexpression of Rpo12 and Rpo11 leads to a prevalent reduction on the cellular abundances of most RNAP subunits. As a control, we also examined RNAP subunit levels in strains overexpressing the large catalytic subunits Rpo1″ and Rpo2″. In contrast to the Rpo12 and Rpo11 strains, these overexpression strains exhibited only mild reductions in the levels of most RNAP subunits, except for slight decreases in Rpo10 and Rpo6 (Fig. 7). These data are consistent with the comparatively minor growth effects caused by the overexpression of Rpo1″ and Rpo2″ (Fig. S6 and Fig. 7A and B), which could possibly have limited effect on the RNAP assembly process.

Thus, the broad reduction in RNAP subunit abundance observed upon Rpo12 and Rpo11 overexpression likely results from the RNAP assembly defects in these strains. Disruption of efficient RNAP assembly could leave many subunits unassembled and, thus, susceptible to proteolytic degradation, leaving to markedly reduced steady-state levels compared to the wild-type strain. This cascade, from stoichiometric imbalance to assembly disruption, subunit depletion, and ultimately impaired growth, provides a unifying explanation for the phenotypes observed in the overexpression strains.

## DISCUSSION

The accurate and efficient assembly of RNA polymerase (RNAP) is fundamental to transcription, the cornerstone of the central dogma, and is essential for cellular survival, adaptation, and development across all domains of life. Despite extensive structural and functional characterization of archaeal RNA polymerase (RNAP), the *in vivo* principles that govern its biogenesis have remained completely unexplored. Here, we present the first comprehensive *in vivo* dissection of the archaeal RNAP assembly pathway. By combining biochemical, genetic, and quantitative approaches, we decipher a modular and hierarchically ordered assembly cascade composed of five key intermediate subcomplexes and reveal a sequential recruitment logic of RNAP subunits *in vivo*. Disrupting the stoichiometric balance, specifically via the overexpression of the small subunits Rpo12 or Rpo11, disrupts assembly, reduces the formation of the holoenzyme, and impairs cellular growth through distinct mechanistic blockades. Additionally, we identify the chaperonin Hsp60 as a potential assembly cofactor co-eluting with RNAP intermediates, supporting a conserved role for chaperone-mediated RNAP biogenesis. Together, our findings reveal that archaeal RNAP biogenesis is a modular and tightly coordinated process that is exquisitely sensitive to subunit stoichiometry. These insights not only fill a longstanding knowledge gap in archaeal transcription biology but also illuminate its evolutionary continuity with bacterial and eukaryotic systems and uncover regulatory vulnerabilities with potential applied relevance.

### *In vivo* assembly model of archaeal RNAP: a mechanistic and evolutionary framework

**Fig 8:**
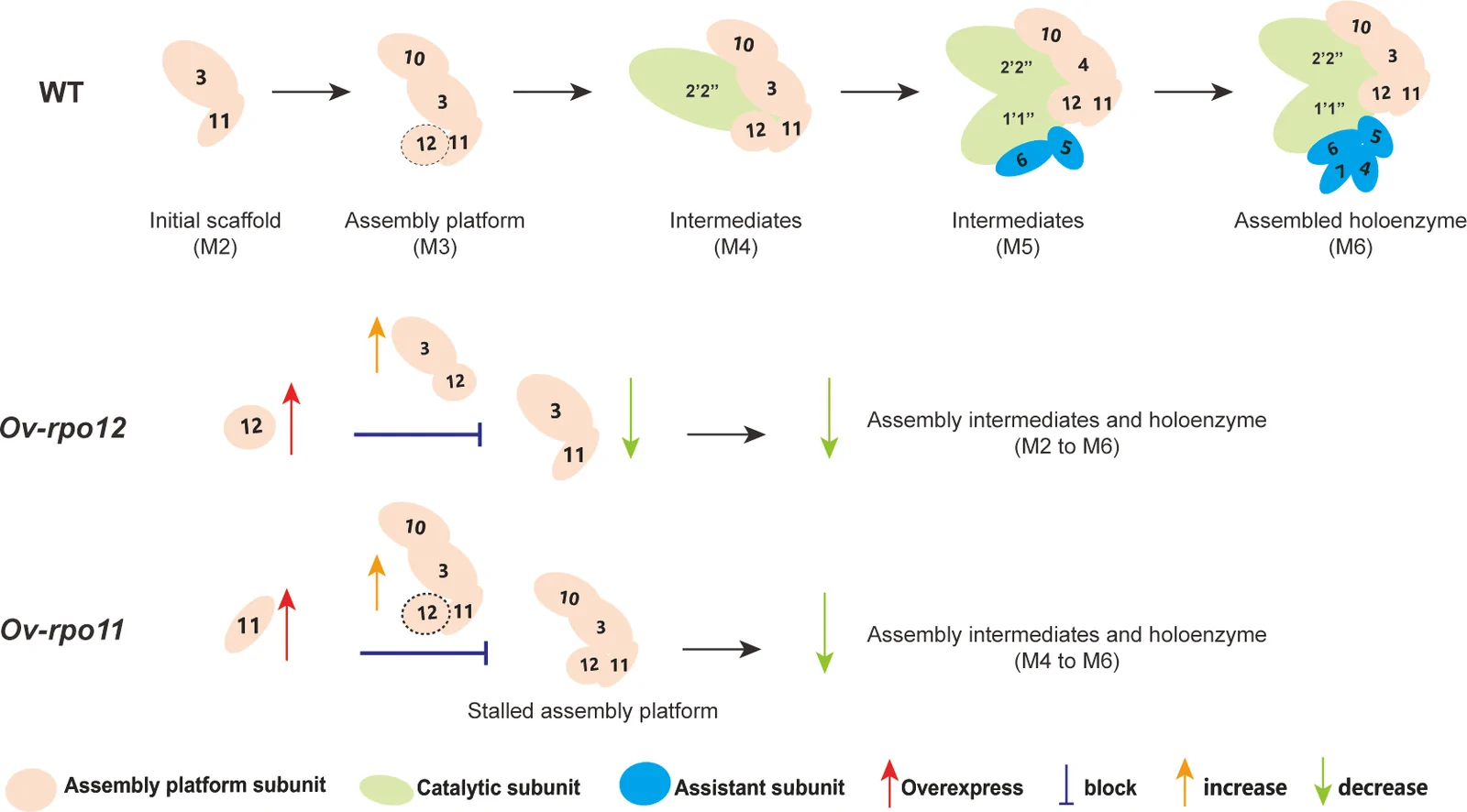
Schematic model of the hierarchical assembly pathway of archaeal RNAP and the effects of subunit stoichiometry imbalance. The assembly of *M. maripaludis* RNAP proceeds through a stepwise pathway involving modular intermediates (M2–M6), culminating in the fully assembled holoenzyme (M6). The Rpo3/11/10/12 subcomplex acts as a central assembly platform for the recruitment of catalytic and accessory subunits. Overexpression of small subunits perturbs this pathway: Rpo12 overexpression (*Ov-rpo12*) causes accumulation of Rpo3/12 subcomplex and reduces abundance of the native Rpo3/11 intermediate, whereas Rpo11 overexpression (*Ov-rpo11*) causes accumulation of the Rpo3/11/10 intermediate. In both cases, these changes coincide with reduced abundance of downstream assembly intermediates and fully assembled holoenzyme, highlighting the importance of balanced subunit stoichiometry for productive RNAP assembly.

Based on the *in vivo* and *in vitro* data delineated in this study, we define five major RNAP assembly intermediates in *M. maripaludis* and reconstruct a sequential *in vivo* assembly cascade (Fig. 8). The assembly initiates with the formation of the Rpo3/11 heterodimer, which acts as an initial scaffold. Then, two additional small subunits, Rpo10 and Rpo12, could be loaded onto Rpo3/11, and consequently, the catalytic subunits Rpo2″ and Rpo2′ could be recruited; while, Rpo3/11 or Rpo3/11/10 may also recruit the catalytic subunit Rpo2″ and Rpo2′ directly without relying on Rpo12 binding. Then, the catalytic subunit Rpo1′1″ and two other small subunits Rpo6 and Rpo5 could be loaded, with the consequent recruitment of Rpo2″; finally, the subunits, Rpo4/7, which form the stalk structure of the RNAP, were additionally loaded to form the fully assembled RNAP holoenzyme. This experimentally grounded assembly map fills a critical gap in our understanding of the archaeal RNAP assembly process and establishes a mechanistic framework for future investigations into RNAP dynamics in archaea. Importantly, it also provides an evolutionary lens to interpret the conserved principles of RNAP biogenesis across the three domains of life.

Comparative analysis reveals that archaeal RNAP assembly shares key features with both bacterial and eukaryotic systems, supporting a deep evolutionary logic. In bacteria, a widely accepted model for RNAP (ααββ′ω subunits) assembly, based on *in vitro* studies, proposes that biogenesis begins with the formation of an αα homodimer, which subsequently recruits the β subunit to form an ααβ intermediate. This intermediate then associates with the β′ subunit, which likely interacts with the ω subunit to stabilize the complex (8, 33). Although nonessential, the ω subunit plays a key role in promoting proper folding and stabilization of the largest catalytic subunit, β′ (6, 33). A strikingly similar modular assembly strategy has been proposed for eukaryotic RNA polymerase II (RNAP II), which retains a homologous ααββ′ω core structure, composed of subunits Rpb3, Rpb11, Rpb2, Rpb1, and Rpb6, respectively (3, 5, 34, 35). These core subunits are conserved across RNAP I, II, and III in yeast. Accordingly, the Rpb3-10-11-12 subassembly complex (corresponding to bacterial αα dimer and archaeal Rpo3-10-11-12) would form and interact with the Rpb2 subassembly complex (Rpb2 and Rpb9, similar bacterial β subunit and archaeal Rpo2′2″) prior to the association with the Rpb1 subassembly module (composed of Rpb1, Rpb5, Rpb6, and Rpb8, similar to bacterial β′ω subunits and archaeal Rpo1′1″-5-6). Rpb6 (homologous to bacterial ω and archaeal Rpo6) would act by stabilizing the largest subunit of RNA pol II for the assembly of RNA pol II in line with the role proposed for the ω subunit (33, 36). Finally, the stalk subcomplex (Rpb4/7) would associate with the preassembled core enzyme (5), although it can dissociate (37). Thus, beyond the known structural and functional homology between archaeal RNAP and eukaryotic RNAP II (e.g., subunit composition, domain organization, and catalytic mechanisms) (3), our data support a modular and hierarchical assembly logic for archaeal RNAP that parallels RNAP II assembly. Particularly notable is the parallel between the archaeal Rpo3/10/11/12 scaffold and the eukaryotic Rpb3/10/11/12 subassembly, as both appear to serve as early organizational platforms for subsequent recruitment of catalytic subunits. This correspondence strongly supports evolutionary continuity in RNAP assembly logic between archaea and eukaryotes. The comparison is also consistent with earlier urea-based disassembly studies of eukaryotic Pol II, which identified stable core intermediates and supported a modular assembly model. Our archaeal Rpo3/12–Rpo2′2″ subassembly appears conceptually analogous, although not identical in composition, to these eukaryotic disassembly intermediates (5, 38). These assembly parallels extend beyond shared components to include stepwise, subcomplex-based biogenesis strategies, underscoring a deeper level of evolutionary conservation than previously appreciated. Such mechanistic resemblance further supports the view that the archaeal RNAP assembly pathway represents an ancestral, streamlined prototype of the more elaborate eukaryotic RNAP II biogenesis process.

Our findings, therefore, suggest that archaeal RNAP assembly not only exemplifies a modular and efficient strategy for constructing a multi-subunit enzyme but also reflects a conserved evolutionary prototype from which the eukaryotic RNAP assembly pathway may have originated. The conservation of intermediate subcomplexes and assembly order across domains strongly supports the hypothesis that the last universal common ancestor (LUCA) employed a similar modular assembly logic for RNAP biogenesis. In this evolutionary context, the archaeal system serves as both a mechanistic bridge and a molecular relic, linking bacterial simplicity with eukaryotic complexity. This perspective enriches our broader understanding of transcriptional evolution and highlights archaeal RNAP biogenesis as a pivotal model for decoding the emergence and architectural logic of gene expression systems across distinct domains of life.

### Stoichiometric regulation as a critical checkpoint

A further advance of this work is the demonstration that archaeal RNAP assembly is exquisitely sensitive to subunit stoichiometry, representing a new regulatory layer underlying archaeal RNAP biogenesis. Overexpression of Rpo12 or Rpo11 did not simply increase their incorporation into mature RNAP, but instead altered early Rpo3-containing assembly-state distribution and reduced downstream assembly and holoenzyme formation. These findings suggest that productive RNAP assembly depends on balanced partitioning of early assembly platforms rather than on the abundance of individual subunits alone. The associated reduction in RNAP assembly states and cellular growth further indicates that assembly-state balance of RNAP subunits functions as an intrinsic regulatory layer linking subunit expression to transcriptional capacity and cellular fitness.

Notably, such stoichiometric sensitivity is not unique to archaea but could represent a conserved regulatory logic across the domains of life. In eukaryotes, for instance, the steady-state level of the small RNAP subunit Rpb10 (a homolog of archaeal Rpo10) is strictly regulated by the RNA-binding protein Rbs1 and the helicase Upf1, which controls Rpb10 mRNA stability and translation. This regulatory mechanism to control Rpb10 at a precise concentration is essential for ensuring correct α_2_β-like module formation during co-translational assembly of RNAP I and RNAP III complexes (39, 40). In bacteria, stoichiometric control is similarly reinforced through autogenous regulation of the *rpoBC* operon, encoding the β and β′ subunits. Feedback inhibition mediated by the RNAP holoenzyme or intermediate α₂β complexes modulates *rpoBC* transcription and translation, ensuring coordinated production of catalytic subunits (41). Classic studies in *E. coli* have demonstrated that imbalanced RNAP subunit expression, particularly the uncoupling between β/β′ and α subunits, has been frequently observed in temperature-sensitive lethal mutants of *E. coli* (41), and temperature-sensitive lethal mutations in the β (RpoB) and β′ (RpoC) can perturb subunit balance, leading to the accumulation and rapid degradation of unassembled intermediates, while the α subunit remains stable due to its role in initiating assembly (41–44). A related imbalance was also observed in *M. maripaludis*. Upon Rpo12 or Rpo11 overexpression, altered early scaffold subunit Rpo3-containing subcomplexes (Rpo3/12 and Rpo3/11/10) accumulated, whereas downstream RNAP assembly intermediates and the assembled holoenzyme were markedly reduced, possibly reflecting impaired assembly progression and/or degradation of unassembled subunits. Thus, in contrast to the nearly stoichiometric subunit levels observed in the wild-type strain (Fig. 5), while both Rpo12 and Rpo11 overexpressed strains exhibited clear subunit abundance imbalances (Fig. 7B). Moreover, our previous identification of an ioTTS-mediated precise control of Rpo12 expression in *M. maripaludis* parallels this internal termination-mediated regulation of the *rplJL–rpoBC* operon (27, 41).

Taken together, our results underscore that archaeal RNAP assembly is not only a structurally ordered process but also a tightly regulated one, integrating mechanisms of subunit-level control, assembly feedback, and degradation surveillance. These findings reinforce the idea that coordinated expression and stoichiometry of RNAP subunits are fundamental and conserved principles underpinning transcriptional machinery across all domains of life.

### Discovery of potential RNAP assembly cofactor and evolutionary conservation of auxiliary systems

In addition to revealing a modular assembly pathway and stoichiometry-sensitive control of archaeal RNAP biogenesis, our study identified candidate auxiliary factors that may assist this process. The MS analysis reproducibly detected the group II chaperonin Hsp60 co-eluting with multiple RNAP assembly intermediates, suggesting a potential association with archaeal RNAP biogenesis. This observation raises a broader conceptual point regarding the assembly of double-psi β-barrel (DPBB) RNA polymerases across evolution. In *M. maripaludis*, the overall *in vivo* assembly trajectory inferred here closely parallels the pathway that was previously reconstructed *in vitro* for archaeal RNAP (26), suggesting that the core assembly trajectory is largely specified by intrinsic subunit compatibility. In this context, Hsp60 may enhance folding efficiency, suppress mis-assembly, or stabilize vulnerable intermediates rather than fundamentally dictating assembly order. Thus, archaeal RNAP appears to retain strong self-organizing capacity while still potentially benefiting from chaperone-assisted quality control *in vivo*.

This may imply an obvious contrast to eukaryotic DPBB RNAPs, whose biogenesis appears to depend much more strongly on dedicated chaperones and assembly factors. In bacteria, chaperones like GroEL support the proper folding of RNAP subunits, particularly β′, and assist in their integration into the core enzyme (45, 46). In eukaryotes, RNAP II biogenesis requires a broader network of auxiliary cofactors, including the heat shock protein Hsp90, members of GPN-loop GTPases, Gpn1 (Npa3), Gpn2, Gp3m, an unconventional prefoldin protein Bud27, and the assembly/import factor Iwr1, which coordinate folding, protection, transport, nuclear import, and quality control in a spatially compartmentalized intracellular environment (47–51). Archaeal RNAP may, therefore, represent an ancestral and comparatively streamlined assembly system, positioned between bacterial self-assembly and the highly cofactor-dependent eukaryotic pathway.

Additionally, our data suggest that archaeal Rpo6 is recruited during late assembly together with Rpo1′-containing intermediates. Given its structural homology to bacterial ω and eukaryotic Rpb6, archaeal Rpo6 may function analogously in stabilizing the largest subunits and promoting productive integration into the holoenzyme. Notably, bacterial ω subunit facilitates β′ folding and prevents aggregation (33, 52), a role that parallels proposed functions of eukaryotic Rpb6 and may extend to archaeal Rpo6.

Together, these parallels suggest that RNAP biogenesis across all domains of life relies on both ordered subunit recruitment and auxiliary systems that safeguard assembly fidelity. More broadly, archaeal RNAP provides a valuable model for understanding how intrinsic self-assembly principles and cofactor-assisted quality control were integrated during the evolution of increasingly complex transcription machineries.

### Implications for targeting archaeal RNAP in methane mitigation strategies

Methane is the second most important greenhouse gas after carbon dioxide, contributing to approximately 20% of the global warming effect since the industrial revolution. Atmospheric methane levels have more than doubled in the last two centuries, with current global emissions estimated at 500–600 Tg annually. Notably, at least ~70% of methane emissions originate from biological methanogenesis, a process carried out exclusively by methanogenic archaea in diverse anaerobic environments (53, 54).

Our delineation of the archaeal RNAP assembly pathway uncovers previously unknown vulnerabilities in methanogenic archaea that may be exploitable for methane mitigation. The discovery that the overexpression of Rpo12 and Rpo11, two small, archaeal-specific RNAP subunits, disrupts RNAP assembly and impairs transcriptional capacity in the model methanogen suggests a new molecular vulnerability of the transcriptional machinery in methanogenic archaea. These subunits could represent novel targets for the development of small molecules or structural mimetics designed to interfere with RNAP assembly in methanogen. Conceptually, such compounds could act by mimicking the overabundant subunit state, sequestering key partners (e.g., Rpo3 in the case of Rpo12) into nonproductive complexes, and thereby halting RNAP assembly. This targeted disruption would be expected to inhibit transcription and growth in methanogens, ultimately suppressing methane production. As these effects arise from interference in early-stage RNAP assembly rather than direct active site inhibition, this strategy may minimize resistance development and off-target effects in non-archaeal species.

Thus, the archaeal RNAP system unveiled in this study could provide a valuable platform for the rational design of transcription-inhibiting agents tailored for methanogenic archaea. The translational potential of this research extends to environmental biotechnology and climate policy, where biologically informed methane mitigation strategies are urgently needed.

### Conclusion

This study presents the first comprehensive model of *in vivo* RNA polymerase assembly in archaea, revealing a modular and hierarchically regulated process supported by conserved subcomplexes and assembly factors. We identify the critical importance of balanced subunit expression, delineate the consequences of subunit dysregulation, and draw evolutionary links with bacterial and eukaryotic systems. Beyond its mechanistic significance, our work provides a conceptual framework for targeting archaeal transcription in methanogenetic archaea, potentially offering a novel biotechnological approach to greenhouse gas mitigation. Together, these findings expand our understanding of archaeal cell biology and position RNAP assembly as a central node in both fundamental and applied microbiology.

## MATERIALS AND METHODS

### Strains, plasmids, and growth conditions

All archaeal strains used in this study are derived from *M. maripaludis* S0001 and listed in Table S5. *M. maripaludis* S0001 and its derivatives were routinely cultivated anaerobically in pre-reduced MCF medium under a gas phase of N_2_/CO_2_ (80:20) at 37°C or 22°C, respectively, as previously described (31, 32). For solid media, 1.5% (wt/vol) agar was added. All recombinant plasmids were generated by Gibson assembly using pMEV4 as the backbone vector and verified by restriction digestion and Sanger sequencing. Promoter sequences and resistance markers are described in Table S6. For genetic selection, puromycin (2.5 g/mL), neomycin (1.0 mg/mL) was used unless otherwise specified. Plasmid construction was carried out in *Escherichia coli* DH5α for cloning and in BL21 (DE3)/pLysS for protein expression. *E. coli* strains were grown in LB medium at 37°C with shaking at 200 rpm. Ampicillin (100 μg/mL) or kanamycin (50 μg/mL) was added as appropriate. The growth of both *M. maripaludis* and *E. coli* strains was monitored by measuring optical density at 600 nm (OD₆₀₀) using a UV-visible spectrophotometer (Thermo Fisher Scientific).

### Size exclusion chromatography

SEC profiling assays were performed as previously described (27, 55). In brief, log-phase cells of *M. maripaludis* cells were collected and resuspended in SEC buffer (150 mM NaCl, 20 mM MgCl₂·6H₂O, 5% glycerol, and 20 mM HEPES at pH 7.5). The cells were lysed by sonication, and then the supernatant was obtained by centrifugation at 4°C and 12,000 × *g* for 30 min. Then, the supernatant was treated with DNase I (100 units) and RNase A (100 units) and incubated at 37°C for 30 min. Five-hundred microliters of the pretreated supernatant was loaded on a Superdex 200 10/300 GL chromatography column (Cytiva), and the eluted fractions were obtained by following the manufacturer’s procedure. Similarly, the roughly purified RNAP complex and subcomplexes, purified using the methods described below (Purification of RNAP complex and subcomplexes from *M. maripaludis*), were applied to a Superdex 200 10/300 GL chromatography column (Cytiva) in independent run to obtain the SEC fractions of the purified RNAP holoenzyme and intermediates as well. Elution was performed at a flow rate of 0.5 mL/min, and in total 48 fraction with 0.5 mL for each were collected. The molecular weight of each SEC fraction was determined by determining a standard curve based on the elution volumes of gel filtration molecular weight markers (Kit No.: MWGF1000 of Sigma-Aldrich). Finally, each SEC fraction was collected for protein identification by SDS-PAGE or Western blotting analysis.

### Expression and purification of recombinant RNAP subunits

His- or GST-tagged RNAP subunits of *M. maripaludis* were expressed in *E. coli* BL21 (DE3)/pLysS from pET28a or pGEX vectors constructed via Gibson assembly using the ClonExpress Multi-Step Cloning Kit (Vazyme). The open reading frames (ORFs) of His-tagged Rpo1″, Rpo2″, Rpo10, Rpo12, Rpo6, Rpo5 proteins, and GST-tagged Rpo4, Rpo7, Rpo3, and Rpo11 proteins were separately cloned into the pET28a or pGEX 6P-1/pGEX 4T-1 expression vectors using Gibson assembly. The recombinant plasmids (Table S5) were verified by restriction digestion and Sanger sequencing and then transformed into *E. coli* BL21 (DE3) pLysS. Positive transformants were screened and cultured at 37°C in LB broth containing either 50 μg/mL kanamycin or 100 μg/mL ampicillin. When the OD_600_ reaches 0.6–0.8, isopropyl-D-thiogalactoside (IPTG) (0.5 mM) was added to induce recombinant protein expression by incubating the cells at 22°C for 16–18 h. Cells were harvested by centrifugation and stored at −80°C. The collected cells were resuspended in binding buffer (His-Tag: 0.5 M NaCl, 20 mM imidazole, 5% glycerol, and 20 mM HEPES at pH 7.5; GST-Tag: 0.5 M NaCl, 20 mM Tris-base, 1 mM DTT, 5% glycerol, pH 7.5) and lysed by sonication, and the supernatant was collected after centrifugation. Then, the supernatant was loaded onto the His-Trap HP or GST-Trap HP (Cytiva) on an ÄKTA purifier following the manufacturer’s procedures. Eluted fractions were analyzed by SDS-PAGE, and protein concentration was determined using a BCA protein assay kit (Thermo Scientific).

### Western blotting assays and quantitative analysis of RNAP subunits

Western blotting was performed to determine the target RNAP subunits present in the SEC fractions and their cellular abundances in *M. maripaludis*. The polyclonal rabbit antisera against each RNAP subunit were generated by immunizing rabbits with purified recombinant RNAP subunits as antigens (BioEasy, China). Logarithmic cells of *M. maripaludis* were harvested, resuspended in lysis buffer (150 mM NaCl, 20 mM MgCl_2_·6H_2_O, 5% [wt/vol] glycerol, and 20 mM HEPES at pH 7.5), and lysed by sonication. The cell lysate was then centrifuged at 14,000 × *g* for 15 min at 4°C. The supernatant was separated by SDS-PAGE (4%–20%) and transferred to a nitrocellulose membrane. Primary antisera of each RNAP subunit were applied at 1:5,000 or 1:2,000 depending on antibody performance, followed by horseradish peroxidase (HRP) conjugated secondary antibodies (1:5,000). The immunoreactive bands were visualized using ECL ultra-sensitive chemiluminescent substrate (Vazyme).

The intracellular concentrations of RNAP subunits were determined by quantitative Western blotting using the subunit-specific antisera described above together with purified recombinant proteins as calibration standards. His- or GST-tagged RNAP subunits were expressed and purified from *E. coli* BL21DE3(pLysS). Serial dilutions of each purified protein were electrophoresed and detected by Western blotting to establish standard curves correlating signal intensity with protein concentration. Cell-free extract of *M. maripaludis* was analyzed in parallel under identical electrophoresis and Western blotting conditions. Band intensities were quantified using ImageJ, and intracellular protein concentrations (pmol/μg total protein) were calculated for each subunit relative to the corresponding standard curves. However, as the antisera were generated against affinity-tagged recombinant proteins and calibrated using the same tagged standards, the resulting values may be influenced by tag-dependent antibody recognition and should be interpreted as apparent abundance estimates rather than precise absolute concentrations. Moreover, differences in antibody sensitivity and epitope accessibility also preclude direct comparison of raw signal intensities among different RNAP subunits.

To determine the distribution of RNAP subunits across SEC fractions, 20–30 µL of each SEC fraction was parallelly loaded on SDS-PAGE (4%–20%) alongside a pre-fractionation cell-free extract serving as a reference for identifying the target subunit. Protein levels in each fraction were quantified by densitometric analysis using ImageJ, and relative abundances were calculated by normalizing the signal intensity of each fraction to the corresponding band in the cell-free extract. For comparative analyses of RNAP assembly profiles, the WT SEC–Western blot data set was used as a common reference and is presented in Fig. 1 and 5 and 6. Identical WT data were reproduced in the latter figures solely to facilitate direct comparison with the corresponding overexpression strains.

### Purification of RNAP complex and subcomplexes from *M. maripaludis*

To purify RNAP complex and its assembly intermediates from *M. maripaludis*, a His10×-tag was fused to the C-terminus of the chromosomal *rpo11* gene in *M. maripaludis* S0001 using a similar approach as described previously (55). Briefly, a His10×-tag was first fused to the C terminus of *Mmp*-Rpo11 via PCR amplification and cloned into pMD19-T to construct the plasmids pMD19-T-*rpoL*-His10×. The puromycin resistance cassette in the plasmid pIJA03 was amplified and overlapped fused to the ~800 bp fragment downstream of *Mmp*-Rpo11. The overlapped PCR fragment was then inserted downstream of *Mmp*-Rpo11-His10× to obtain pMD19-T-*rpo11*-His10×-*pac*. Then, DNA fragment was amplified from this plasmid and transformed into *M. maripaludis* S0001 to obtain His10× tagged *Mmp*-Rpo11 strain (Table S5). The resulting strain was cultured at 37°C to the logarithmic phase (OD_600_ = 0.4–0.6) and then harvested by centrifugation. Cell pellets were resuspended in binding buffer (0.5 M NaCl, 20 mM imidazole, 5% glycerol, and 20 mM HEPES at pH 7.5) supplemented with protein inhibitors cocktail (Mei5bio, China). The supernatant was obtained by centrifugation (14,000 × *g*, 15 min, 4°C), treated with DNase I and RNase A (100 U each, 30 min, 37°C), and loaded onto a His-Trap HP column (Cytiva) for purification. For purifying both RNAP complex and its intermediates, the His-trap column was gently washed using wash buffer I (0.5 M NaCl, 50 mM imidazole, 5% glycerol, and 20 mM HEPES at pH 7.5) for 10 column volumes, while for strictly purifying pure RNAP complex but without the assembly intermediates, the His-trap column was strictly washed using wash buffer II (1 M NaCl, 50 mM imidazole, 5% glycerol, and 20 mM HEPES at pH 7.5) for at least 30 min. Elution was performed by gradient imidazole concentration from 50 to 500 mM on an ÄKTA purifier (GE Healthcare) with buffer (0.5 M NaCl, 500 mM imidazole, 5% glycerol, and 20 mM HEPES at pH 7.5). Eluted proteins were analyzed by SDS-PAGE, concentrated using Amicon Ultra-15 centrifugal filters (30 kDa MWCO, Millipore), and then loaded onto a Superdex 200 10/300 GL chromatography column (Cytiva) for the subsequent fraction profiling of RNAP holoenzyme and intermediates. The SEC fractions were then analyzed using SDS-PAGE combined with Western blotting, and the main intermediate components were cut and identified by mass spectrometry.

### Mass spectrometry analysis of RNAP subcomplexes and potential associating cofactors

SDS-PAGE-separated protein bands corresponding to RNAP subcomplexes were excised and used for mass spectrometry (MS) analysis as described previously (56). First, the excised SDS-PAGE gel band was washed with 20% methanol and blocked with 0.1% Tween-20. Samples were reduced with 10 mM DTT, alkylated with 55 mM iodoacetamide, and subjected to in-gel tryptic digestion overnight at 37°C. The digestion mixtures were centrifuged, and supernatants were freeze-dried and reconstituted in 0.1% formic acid for MS analysis.

Peptides were analyzed on a Q Exactive Plus mass spectrometer (Thermo Scientific) coupled to an EASY-nLC 1200 system. MS/MS data were processed using MaxQuant (v1.6) against mapping the peptide sequences against the *M. maripaludis* S2 genome. Proteins identified with ≥2 unique peptides and an FDR <1% were considered confidently assigned. RNAP subunit composition and consistently co-purified interacting proteins were analyzed to identify assembly intermediates and potential cofactors. All mass spectrometry analyses were performed by the Proteomics Core Facility of the Institute of Microbiology, Chinese Academy of Sciences (Beijing, China).

### Disassembly assay of RNAP complex

To assess the stability and composition of RNAP subcomplex, the strictly purified RNAP holoenzyme was first loaded onto self-filled Ni-NTA column by packing an empty column with a metal chelation chromatography medium (Ni-NTA QZT 6FF, Shenhui Microsphere Tech, China). After binding, the column-bound RNAP complex was subjected to increasing concentrations of urea (0–5 M) in binding buffer (0.5 M NaCl, 20 mM imidazole, 5% glycerol, and 20 mM HEPES at pH 7.5), and the samples were incubated at 4°C for 1 h. Furthermore, the sample was washed by binding buffer with different salt concentrations when required and finally eluted by elution buffer (0.5 M NaCl, 500 mM imidazole, 5% glycerol, and 20 mM HEPES at pH 7.5). Eluted fractions were analyzed by SDS-PAGE, and the gels were analyzed by Coomassie brilliant blue or silver staining. The disassembly profiles of RNAP subunits were used to infer interaction strength and assembly hierarchy.

### Construction of RNAP subunit overexpression strains

To generate overexpression strain constructs for RNAP subunits, open reading frames (ORFs) of *rpo12*, *rpo11*, *rpo1′rpo1″*, *rpo2′rpo2″*, *rpo10*, *rpo7*, *rpo4*, *rpo6*, and *rpo5* were PCR-amplified from *M. maripaludis* genomic DNA using primer pairs listed in Table S6. The amplified fragments were cloned into the expression vector pMEV4 under the control of the constitutive promoter P_0386_, yielding plasmids of the form pMEV4-P_0386_-*rpoX-pac* (Table S5). The plasmid construction was verified by DNA sequencing and then transformed into *M. maripaludis* using polyethylene glycol (PEG)-mediated transformation, and transformants were selected on puromycin (2.5  μg/mL) as described previously (31). A plasmid expressing the mCherry gene under identical regulatory elements was used as a control. Overexpression strains are listed in Table S5.

## Data Availability

The mass spectrometry proteomics data have been deposited to the ProteomeXchange Consortium via the PRIDE partner repository with the data set identifier PXD081683.

## SUPPLEMENTAL MATERIAL

Supplemental material mbio.01440-26-s0001.docx Figures S1 to S6; Tables S1 to S6.

Data sets S1-S4 mbio.01440-26-s0002.xlsx LC-MS/MS identification of RNAP subunits and candidate assembly cofactors from SEC fractions across all three biological replicates.

ASM does not own the copyrights to Supplemental Material that may be linked to, or accessed through, an article. The authors have granted ASM a non-exclusive, world-wide license to publish the Supplemental Material files. Please contact the corresponding author directly for reuse.
