# Supplementary material for: Dissection of in vivo archaeal RNA polymerase assembly reveals a modular pathway and stoichiometric control
Source: mBio. 2026 Jul 28;17(9):e01440-26. doi: 10.1128/mbio.01440-26 (PMC13556214; doi:10.1128/mbio.01440-26)
Supplement: Supplementary file 1 [file mbio.01440-26-s0001.docx]

**Supplementary Information for**

**Dissection of *In Vivo* Archaeal RNA Polymerase Assembly Reveals a Modular Pathway and Stoichiometric Control**

Jie Li ^1,2, #, *^, Wen Qi ^1,2, #^, Huaping Duan ^1, 2, #^, Derong Ren^1,2, #^, Jingfang Liu ^1, 2^, Xiuzhu Dong ^1,2^

1, State Key Laboratory of Microbial Diversity and Innovative Utilization, Institute of Microbiology, Chinese Academy of Sciences, Beijing 100101, China

2, University of Chinese Academy of Sciences, No.19A Yuquan Road, Shijingshan District, Beijing 100049, China

*, Correspondence to: **Jie Li**, No.1 Beichen West Road, Beijing 100101. Tel. 86-10-6480 7567. Email: [lijie824@im.ac.cn](mailto:lijie824@im.ac.cn).

^#^ These authors contributed equally.

**This file contains:**

**Supplementary Figure S1 to S6.**

**Supplementary Tables S1 to S6.**

**Figure S1. Calibration curve for SEC used in RNAP subcomplex analysis.**

Standard curve plotting the logarithm of molecular weight (kDa) of reference proteins against the ratio of their elution volume (Ve) to the column void volume (V₀). This calibration was used to estimate the apparent molecular weights of RNAP assembly intermediates in SEC experiments.

**Figure S2 AlphaFold2-predicted structural modular composed of Rpo6, Rpo5, Rpo1′, and Rpo1″.**

Structure prediction suggests that Rpo6 and Rpo5 form a stable interaction interface with Rpo1′ and Rpo1″, supporting the hypothesis that Rpo6 and 5 are possibly co-recruited via forming a pre-assembled subcomplex by association with Rpo1′ during late-stage RNAP assembly.

**Figure S3. Mass spectrometry analysis reveals the subunit composition of *M. maripaludis* RNAP assembly intermediates.**
Bar chart showing MaxQuant score values for identified RNAP subunits in each indicated SEC fraction subjected to mass spectrometry. Higher scores indicate greater confidence in subunit identification. The identified RNAP subunits with their theoretical molecular weights are indicated with corresponding colors.

**Figure S4. SDS–PAGE analysis of purified *M. maripaludis* RNAP holoenzyme.**

Coomassie-stained SDS–PAGE gel of purified RNAP holoenzyme under native (non-denaturing) conditions. RNAP subunits are indicated by arrows via reference to the protein markers.

**Figure S5.** **Standard curves for quantitative Western blot analysis of RNAP subunits.**

Linear standard curves were generated by plotting the known concentrations of purified recombinant RNAP subunits against the corresponding band intensities detected by Western blotting. These curves were used to determine the absolute intracellular abundance of RNAP subunits in *M. maripaludis*.

**Figure S6. Growth analysis of *M. maripaludis* strains overexpressing individual RNAP subunits.**

(A) Growth curves of RNAP subunit overexpression strains cultured at 22 °C.

(B) Growth curves of the same strains cultured at 37 °C.

Growth was monitored by measuring OD_600_ at regular intervals over time.

Growth curves of RNA polymerase subunit overexpressed strains measured at 22 °C and 37 °C.

**Table S1 Conserved RNA polymerase (RNAP) subunits across three domains of life.**

| **Bacteria** | **Archaea** | **Eukaryotes** | | |
| --- | --- | --- | --- | --- |
|  |  | RNAP II | RNAP I | RNAP III |
|  | | | | |
| β-subunit | Rpo1 (RpoA) | RPB1 | A190 | C160 |
| β-subunit | Rpo2 (RpoB) | RPB2 | A135 | C128 |
| α-subunit | Rpo3 (RpoD) | RPB3 | AC40 | AC40 |
| α-subunit | Rpo11 (RpoL) | RPB11 | AC19 | AC19 |
| ω-subunit | Rpo6 (RpoK) | RPB6 | RPB6 | RPB6 |
|  | Rpo5 (RpoH) | RPB5 | RPB5 | RPB5 |
|  | Rpo8 (RpoG) | RPB8 | RPB8 | RPB8 |
|  | Rpo10 (RpoN) | RPB10 | RPB10 | RPB10 |
|  | Rpo12 (RpoP) | RPB12 | RPB12 | RPB12 |
|  | Rpo4 (RpoF) | RPB4 | A14 | C17 |
|  | Rpo7 (RpoE) | RPB7 | A43 | C25 |
|  |  |  |  | C11 |
|  | Rpo13 |  |  |  |

**Table S2 The molecular weight, amino acid length, isoelectric point, and elution volume of each RNA polymerase subunit.**

| **Subunits** | **Theoretical molecular weight (Da)** | **Amino acid length** | **isoelectric points** | **Theoretical elution volume (mL)** |
| --- | --- | --- | --- | --- |
| Rpo1′′ | 42795.33 Da | 386 | 5.13 | 16.89mL |
| Rpo2′′ | 55882.16 Da | 499 | 5.78 | 16.21mL |
| Rpo3 | 20137.91 Da | 180 | 4.58 | 18.81mL |
| Rpo11 | 10828.44 Da | 96 | 4.59 | 20.39mL |
| Rpo10 | 8269.42 Da | 68 | 5.09 | 21.08mL |
| Rpo12 | 5184.33 Da | 46 | 9.81 | 22.27mL |
| Rpo1′ | 98845.89 Da | 889 | 6.15 |  |
| Rpo2′ | 68572.02 Da | 611 | 5.57 |  |
| Rpo6 | 6186.19 Da | 57 | 8.89 | 21.82mL |
| Rpo5 | 8782.33 Da | 78 | 5.57 | 20.93mL |
| Rpo7 | 21030.09 Da | 187 | 4.76 | 18.70mL |
| Rpo4 | 12381.13 Da | 107 | 4.46 | 20.05mL |

**Table S3 The theoretical molecular weight, isoelectric point, and subunit composition of RNA polymerase intermediate complexes M1~M6 are presented.**

| **Intermediates** | **component** | **Theoretical molecular weight (Da)** | **isoelectric points** | **Theoretical elution volume (mL)** |
| --- | --- | --- | --- | --- |
| M1 | Rpo7-Rpo4 | 33393.21 Da | 4.62 | 17.79 mL |
| M2 | Rpo3-Rpo11 | 30948.34 Da | 4.58 | 18.00 mL |
| M3 | Rpo3-Rpo11-Rpo10 | 39199.75 Da | 4.67 | 17.37 mL |
| M4 | Rpo3-Rpo11-Rpo10-Rpo2**′**-Rpo2**′′** | 155366.49 Da | 5.30 | 13.74 mL |
| M5 | Rpo3-Rpo11- Rpo10-Rpo12- Rpo2**′**-Rpo2**′′**-Rpo1**′**-Rpo1**′′**- Rpo6-Rpo5 | 325321.89 Da | 5.60 | 11.80 mL |
| M6 | Rpo3-Rpo11-Rpo2**′**-Rpo2**′′**-Rpo1**′**-Rpo1**′′**-Rpo10-Rpo12-Rpo6-Rpo5-Rpo7-Rpo4 | 358697.09 Da | 5.43 | 11.54 mL |

**Table S4 The total proportion of each intermediate complex (M1-M6) in the Western blot bands for the wild-type RNA polymerase subunits (Rpo10, Rpo3, Rpo2′′, and Rpo4).**

| **Subunits** | **M1** | **M2** | **M3** | **M4** | **M5** | **M6** |
| --- | --- | --- | --- | --- | --- | --- |
| Rpo2′′ | 3.35% | 4.36% | 4.75% | 8.41% | 18.97% | 59.70% |
| Rpo3 | 2.80% | 5.06% | 14.26% | 9.33% | 13.82% | 54.97% |
| Rpo10 | 0.59% | 0.60% | 18.82% | 3.32% | 25.90% | 47.63% |
| Rpo4 | 19.06% | 18.55% | 14.24% | 0.76% | 3.42% | 46.87% |

**Table S5 The strains and plasmids used in this study.**

| Strains and plasmids | Characteristics and descriptions | Reference or  sources |
| --- | --- | --- |
| **Strain** | | |
| *E. coli*  BL21(DE3)pLysS | F-ompT hsdS(rB-mB-) gal dcm(DE3)pLysS Camr | TransGen,  Beijing |
| Mmp-pMEV4-P_0386_-  *mCherry* | pMEV4-*mCherry*, Pur^R^，  S0001 with *mCherry* complement | This study |
| Mmp-pMEV4-P_0386_-  *rpo1′′* | pMEV4-*mCherry*, Pur^R^，  S0001 with *mCherry* complement | This study |
| Mmp-pMEV4-P_0386_-  *rpo2*′′ | pMEV4-*mCherry*, Pur^R^，  S0001 with *mCherry* complement | This study |
| Mmp-pMEV4-P_0386_-  *rpo12* | pMEV4-*mCherry*, Pur^R^，  S0001 with *mCherry* complement | This study |
| Mmp-pMEV4-P_0386_-  *rpo11* | pMEV4-*mCherry*, Pur^R^，  S0001 with *mCherry* complement | This study |
| Mmp-pMEV4-P_0386_-  *rpo10* | pMEV4-*mCherry*, Pur^R^，  S0001 with *mCherry* complement | This study |
| Mmp-pMEV4-P_0386_-  *rpo7* | pMEV4-*mCherry*, Pur^R^，  S0001 with *mCherry* complement | This study |
| Mmp-pMEV4-P_0386_-  *rpo4* | pMEV4-*mCherry*, Pur^R^，  S0001 with *mCherry* complement | This study |
| Mmp-pMEV4-P_0386_-  *rpo6* | pMEV4-*mCherry*, Pur^R^，  S0001 with *mCherry* complement | This study |
| Mmp-pMEV4-P_0386_-  *rpo5* | pMEV4-*mCherry*, Pur^R^，  S0001 with *mCherry* complement | This study |
| *M.maripaludis* S2-*rpoL*-His_10_-tag | S0001 with *rpoL* His_10_-Tag | This study |
| **Plasmids** | | |
| pMEV4-P_0386_-*pac*-*mCherry* | pMEV4 containing promoter of *MMP0386*, *mCherry*, Pur^R^ | This study |
| pMEV4-P_0386_-*pac- rpo1*′′ | pMEV4 containing promoter of *MMP0386*, *MMP1364*(*rpo1′′*), Pur^R^ | This study |
| pMEV4-P_0386_*-pac- rpo2′′* | pMEV4 containing promoter of *MMP0386*, *MMP1361(rpo2′′)*, Pur^R^ | This study |
| pMEV4-P_0386_-*pac-rpo12* | pMEV4 containing promoter of *MMP0386, MMP0248(rpo12)* ORF, Pur^R^ | This study |
| pMEV4-P_0386_-*pac-rpo11* | pMEV4 containing promoter of *MMP0386*, *MMP0261(rpo11)*, Pur^R^ | This study |
| pMEV4-P_0386_-*pac- rpo10* | pMEV4 containing promoter of *MMP0386*, *MMP1326(rpo10)*, Pur^R^ | This study |
| pMEV4-P_0386_-*pac- rpo7* | pMEV4 containing promoter of *MMP0386* *MMP0440(rpo7),* Pur^R^ | This study |

| pMEV4-P_0386_-*pac-rpo4* | pMEV4 containing promoter of *MMP0386, MMP0092(rpo4)*, Pur^R^ | This study |
| --- | --- | --- |
| pMEV4-P_0386_-*pac-rpo6* | pMEV4 containing promoter of *MMP0386, MMP1327(rpo6)* ORF, Pur^R^ | This study |
| pMEV4-P_0386_-*pac-rpo5* | pMEV4 containing promoter of *MMP0386, MMP1360(rpo5)*, Pur^R^ | This study |
| pET28a | Kana^R^ | This study |
| pET28a-*rpo1''* | pET28a with *MMP1364* inserted, Kana^R^ | This study |
| pET28a-*rpo2''* | pET28a with *MMP1361* inserted, Kana^R^ | This study |
| pET28a-*rpo10* | pET28a with *MMP1326* inserted, Kana^R^ | This study |
| pET28a-*rpo12* | pET28a with *MMP0248* inserted, Kana^R^ | This study |
| pET28a-*rpo6* | pET28a with *MMP1327* inserted, Kana^R^ | This study |
| pET28a-*rpo5* | pET28a with *MMP1360* inserted, Kana^R^ | This study |
| pGEX-4T-1 | Amp^R^ | Takara, Japan |
| pGEX-6P-1-*rpo7/4* | pGEX-6P-1 with *MMP 0440/0092* inserted, Amp^R^ | This study |
| pGEX-4T-1-*rpo3/11* | pGEX-4T-1 with *MMP 1322/0261* inserted, Amp^R^ | This study |

**Table S6** The primers used in this study.

| **Primers** | **Sequence（5'-3'）** | **Purpose** |
| --- | --- | --- |
| pET28a-F | CTCGAGCACCACCACCAC | Recombinant protein  purification |
| pET28a-R | GGTATATCTCCTTCTTAAAGTTAAACAAAATTAT  TTCTAGAGGGGAATTGTT | Recombinant protein  purification |
| *rpo2′′*-pET28a-F | GATATACCATGGGCAGCAGCATGGAATCCCGTGTCGTTG | Construction of plasmid  pET28a-*rpo2′′* |
| *rpo2′′*-pET28a-R | CAGTGGTGGTGGTGGTGGTGCTCGAGGTTTTCCTTTGTGAGCCC | Construction of plasmid  pET28a-*rpo2′′* |
| *rpo1′′*-pET28a-F | CTTTAAGAAGGAGATATACCATGCAAATGGCCGATTTAGAAAA | Construction of plasmid  pET28a-*rpo1′′* |
| *rpo1′′*-pET28a-R | AGTGGTGGTGGTGGTGGTGCTCGAGTAATTCTTT  TCCTTCTTCGTAACTCTT | Construction of plasmid  pET28a-*rpo1′′* |
| *rpo10*-pET28a-F | CTTTAAGAAGGAGATATACCGTGATATTTCCAATCAGATGTTTTTCT | Construction of plasmid  pET28a-*rpo10* |
| *rpo10*-pET28a-R | AGTGGTGGTGGTGGTGGTGCTCGAGTTTATATTCGACGATATCATCAAATAATTC | Construction of plasmid  pET28a-*rpo10* |
| *rpo12*-pET28a-F | CTTTAAGAAGGAGATATACCATGGCAGAGTACAAATGTTCCA | Construction of plasmid  pET28a-*rpo12* |
| *rpo12*-pET28a-R | AGTGGTGGTGGTGGTGGTGCTCGAGCTTGCCTGAACTTTTTTAACTAT | Construction of plasmid  pET28a-*rpo12* |
| *rpo6*-pET28a-F | CTTTAAGAAGGAGATATACCATGAAATTAACCAAATTTGAAACTGC | Construction of plasmid  pET28a-*rpo6* |
| *rpo6*-pET28a-R | AGTGGTGGTGGTGGTGGTGCTCGAGATTTTTAGCCTGTTTTTTAACACATAA | Construction of plasmid  pET28a-*rpo6* |
| *rpo5*-pET28a-F | CTTTAAGAAGGAGATATACCGTGAAAATATCGTCACATGAAATGG | Construction of plasmid  pET28a-*rpo5* |
| *rpo5*-pET28a-R | AGTGGTGGTGGTGGTGGTGCTCGAGGAGTGAAGTTGCTATTACTAATCTGT | Construction of plasmid  pET28a-*rpo5* |
| *rpo3*- pGEX-4T-1-F | GGAATTCATGAAAATGGAATTAAAAGCCCC | Construction of plasmid  pGEX-4T-1-*rpo3/11* |

| *rpo3*- pGEX-4T-1-R | GGAATTCTTAATTTTCGTCACTTAATTCTTGGC | Construction of plasmid  pGEX-4T-1-*rpo3/11* |
| --- | --- | --- |
| *rpo11*- pGEX-4T-1-F | CCGCTCGAGGAGGTAATTAAATATGAACTACGTTAAAATCATTG | Construction of plasmid  pGEX-4T-1-*rpo3/11* |
| *rpo11*- pGEX-4T-1-R | CCGCTCGAGTTATAAATCTTCAAGGGTTTTGTTACA | Construction of plasmid  pGEX-4T-1-*rpo3/11* |
| *rpo7*- pGEX-6P-1-F | GGAATTCTGAGGTGGTTATTTGTACAA | Construction of plasmid  pGEX-4T-1-*rpo7/4* |
| *rpo7*- pGEX-6P-1-R | GGAATTCTTAATTTTCTTCACTTAGTGTTT | Construction of plasmid  pGEX-4T-1-*rpo7/4* |
| *rpo4*- pGEX-6P-1-F | CAGGGGCCCCTGGGATCCCCGATGATTGGAAAAGAAATTATTTCC | Construction of plasmid  pGEX-4T-1-*rpo7/4* |
| *rpo4*- pGEX-6P-1-R | TTATTTGAATTTACTAACTACTTCTAAAA | Construction of plasmid  pGEX-4T-1-*rpo7/4* |
| *mCherry*-P_0386_-F | ACTAGAGTGCAGGTAGCGCTATGGTTTCAAAAGGAGAAGAAG | Construction of *mCherry*  expression strain |
| *mCherry*-P_0386_-R | CTGCAGCGGCCGCTACTAGTTTATTTGTATAATTCATC | Construction of *mCherry*  expression strain |
| pMEV4-P_0386_-F | ACTAGTAGCGGCCGCTGC | Construction of  overexpression strains |
| pMEV4-P_0386_-R | AGCGCTACCTGCACTCTAG | Construction of  overexpression strains |
| *rpo1′′*-P_0386_-F | ACTAGAGTGCAGGTAGCGCTATGGATAGATTTGATGTTCCAAAGG | Construction of *rpo1′′*  overexpression strain |
| *rpo1′′*-P_0386_-R | AGTAAAAAAATAAGGTTTTTAGTGGTGGTGGTGGTGGTGTAATTCTTTTCCTTCTTCG | Construction of *rpo1′′*  overexpression strain |
| *rpo2′′*-P_0386_-F | ACTAGAGTGCAGGTAGCGCTATGGAATCCCGTGTCGTTGT | Construction of *rpo2′′*  overexpression strain |
| *rpo2′′*-P_0386_-R | AGTAAAAAAATAAGGTTTTTAGTGGTGGTGGTGGTGGTGTGCTCTGTCTTTTAATTCT | Construction of *rpo2′′*  overexpression strain |
| *rpo12*-P_0386_-F | CTAGAGTGCAGGTAGCGCTATGGCAGAGTACAAATGTTC | Construction of *rpo12*  overexpression strain |
| *rpo12*-P_0386_-R | CTGCAGCGGCCGCTACTAGTTTATCTTGCCTGAACTTTTTTAAC | Construction of *rpo12*  overexpression strain |
| *rpo11*-P_0386_-F | ACTAGAGTGCAGGTAGCGCTATGAACTACGTTAAAATCATTGAA | Construction of *rpo11*  overexpression strain |
| *rpo11*-P_0386_-R | CTGCAGCGGCCGCTACTAGTTTATAAATCTTCAAGGGTTTTGT | Construction of *rpo11*  overexpression strain |
